# Earliest African evidence of carcass processing and consumption in cave at 700 ka, Casablanca, Morocco

**DOI:** 10.1038/s41598-020-61580-4

**Published:** 2020-03-16

**Authors:** Camille Daujeard, Christophe Falguères, Qingfeng Shao, Denis Geraads, Jean-Jacques Hublin, David Lefèvre, Mohssine El Graoui, Mathieu Rué, Rosalia Gallotti, Vincent Delvigne, Alain Queffelec, Eslem Ben Arous, Olivier Tombret, Abderrahim Mohib, Jean-Paul Raynal

**Affiliations:** 1 HNHP-UMR 7194, CNRS, MNHN, UPVD, Sorbonne Universités, Institut de Paléontologie Humaine, 1 rue René Panhard, 75013 Paris, France; 2 College of Geography Science, Nanjing Normal University, 1, Wenyuan Road, 210023 Nanjing, China; 3 CR2P-UMR 7207, CNRS, MNHN, Sorbonne Universités, CP 38, 8 rue Buffon, 75231 Paris cedex 05, France; 4 Department of Human Evolution, Max Planck Institute for Evolutionary Anthropology, Deutscher Platz 6, 04103 Leipzig, Germany; 5 Collège de France, 11 place Marcelin Berthelot, 75005 Paris, France; 6 Université Paul Valéry Montpellier 3, CNRS, UMR 5140 Archéologie des sociétés méditerranéennes, Campus Saint Charles, 34199 Montpellier, France; 7 Institut National des Sciences de l’Archéologie et du Patrimoine (INSAP), Madinat al-Irfane, les Instituts - Hay Riyad, B.P. 6828, 10100 Rabat, Morocco; 8 Paléotime SARL, 6173 avenue JS Achard-Picard, 38250 Villard-de-Lans, France; 9 Université de Bordeaux, CNRS, UMR 5199 PACEA, Bâtiment B2, allée Geoffroy Saint-Hilaire, CS 50023, 33615 Pessac cedex, France; 10 Service de Préhistoire, Université de Liège, place du XX août, 4000 Liège, Belgium; 11 Direction provinciale de la Culture, Avenue Mohammed V, quartier administratif, Kénitra, Morocco

**Keywords:** Evolution, Archaeology

## Abstract

To date, in Africa, evidence for animal processing and consumption in caves routinely used as living spaces is only documented in the late Middle Pleistocene of the North and South of the continent and postdates the Middle Pleistocene in East Africa. Here we report the earliest evidence in a North-African cave (Grotte des Rhinocéros at Casablanca, Morocco) of cut, percussion and human gnawing marks on faunal remains directly associated with lithic knapping activities in the same space and in a well-documented stratified context. Ages for this Acheulean site are provided by the dating of herbivorous teeth to 690–720 ka and 520–550 ka (lower and upper sets) by combined Electron Spin Resonance (ESR) and U-series techniques. Traces of butchery on gazelle, alcelaphin, and zebra bones demonstrate that hominins had primary access to herbivore carcasses. Hominins brought and consumed meat in the cave, as documented by herbivore bones bearing human tooth marks concentrated in a circumscribed area of the excavation. In Africa, this site provides the earliest evidence for *in situ* carcass processing and meat-eating in cave, directly associated with lithic production and demonstrates the recurrent use by early Middle Pleistocene hominins of a North African cave site 400 000 years before that by *Homo sapiens* at Jebel Irhoud (Morocco).

## Introduction

In Africa, animal consumption strategies related to Acheulean industries (1.8–0.4 Ma) are poorly understood in the early and early Middle Pleistocene record^1,2^. Very few sites provided stone tools spatially and functionally associated with the exploitation of fauna in a multi-stratified well-dated context. Besides, all early and early Middle Pleistocene Acheulean sites in Africa are open-air sites, except Wonderwerk cave and Cave of Hearths in South Africa, which yielded Early Stone Age (ESA) industries with no associated cut marked bones^3–5^. Some other Southern African sites with occupations assigned to the early Acheulean are located in the “Cradle of Humankind World Heritage” area. That is the case of Sterkfontein Member 5 West or Swartkrans Members 2 and 3, which have yielded ESA industry and cut marked bones, but either mixed with younger material or not definitely associated, and both are in a pitfall karstic context^6^. The only South African Acheulean site providing cut marked faunal remains recovered in primary association with artefacts, is that of the open-air site of the Elandsfontein dune (~1.0–0.6 Ma)^7^.

Among the oldest Eastern Africa Acheulean open-air sites, the Kokiselei and Konso excavations provided vertebrate remains, some with cut and percussion marks^8–11^. FLK West at Olduvai Gorge (~1.7 Ma) also provided evidence of fauna consumption associated with large-sized tools probably by early *Homo erectus sensu lato*^12^.The slightly younger site of OGS-12 at Gona (~1.6–1.2 Ma) yielded a faunal assemblage with evidence of cut marks (slicing marks) and hominin-induced bone breakage^13^. At Garba IVD (~1.6 Ma) at Melka Kunture, a rich faunal assemblage was recovered together with an early Acheulean industry. Nevertheless, faunal remains are affected by a strong hydraulic action and they do not preserve the original surfaces. Percussion impact points and small percussion flakes are documented, while *striae* produced by lithic tools are almost completely absent^14,15^. Peninj Acheulean sites (~1.5 Ma) have lithic artefacts with some fossil bones resulting from hominid behaviour. It has been argued that the scarcity of fauna in these sites must reflect a behavioral strategy because it cannot be entirely explained through taphonomic constrains^16–18^. At Olduvai Gorge, in Upper Bed II, the BK site (~1.33 Ma) yielded an Acheulean assemblage along with evidence of the exploitation by *Homo erectus s.l*. of big game such as *Pelorovis* and *Sivatherium*. Green bone breakage, percussion notches and cut marks are frequent and their location indicates acquisition by more active strategies than scavenging^19,20^. Finally, in the Okote Member of the Koobi Fora Formation, northern Kenya (~1.6–1.5 Ma), we have abundant evidence of butchery (cut and percussion marks) with no associated stone tools^21–23^.

The long stratigraphic sequences of East Africa include a limited number of sites in the 1.4–0.7 Ma range, several of which are of uncertain age^24–30^. At West Turkana, no sites are known between Kokiselei 4, dated to 1.76 Ma, and Nadung’a 4, dated to ~0.7 Ma^26^. Despite a spatial association of stone tools and bones retrieved from an *in situ* excavation, this latter site yielded artefacts but no butchery marks on carcasses^31^. At Gombore II-2 (~ 0.7 Ma) at Melka Kunture, some cut marks indicate defleshing and disarticulation of hippopotamus carcasses^32,33^. At Olduvai, only JK^34^ in Bed III yielded substantial concentrations of fauna and stone artefacts.

The East African sites of the late Early/early Middle Pleistocene are either devoid of fauna or, when present, fossils are not definitely associated with stone tools. This is also true when Acheulean technology appeared in North Africa, where most discoveries lack a stratigraphic context^35–42^. As regards the Early/Middle Pleistocene, the only known North African Acheulean sites providing industries and associated faunal remains in a stratigraphic context are located in Maghreb: Tighennif (~1 Ma, Algeria) and level L of Thomas Quarry I (~1.2–0.8 Ma, Morocco). Unfortunately, given the lack of taphonomic data in the former site, and the bad bone preservation in the latter, little is known about butchery and carcass processing for that period^43–45^.

Acheulean industry, human and faunal remains are documented in the more recent deposits of the ‘Grotte à Hominidés’ of Thomas Quarry I^46,47^. However, taphonomic analyses have shown that the carnivores were the main agents of faunal accumulation, including that of some of the human remains, as both ends of a human femoral shaft fragment present notches, polishing and tooth marks left by a hyena^48^. Later, in North Africa, in the Middle Pleistocene cave site of Jebel Irhoud, there is evidence of *in situ* processing of herbivore carcasses, associated with little lithic on-site production and fire use, indicating prolonged occupation and hunting activities^49^.

Here we report on the first find of an *in situ* cave assemblage of cut, percussion and human gnawing marks on faunal remains directly associated with a rich Acheulean industry at Grotte des Rhinocéros (GDR), Oulad Hamida 1 Quarry, dated to c. 700 ka.

## Site Presentation

**Figure 1:**
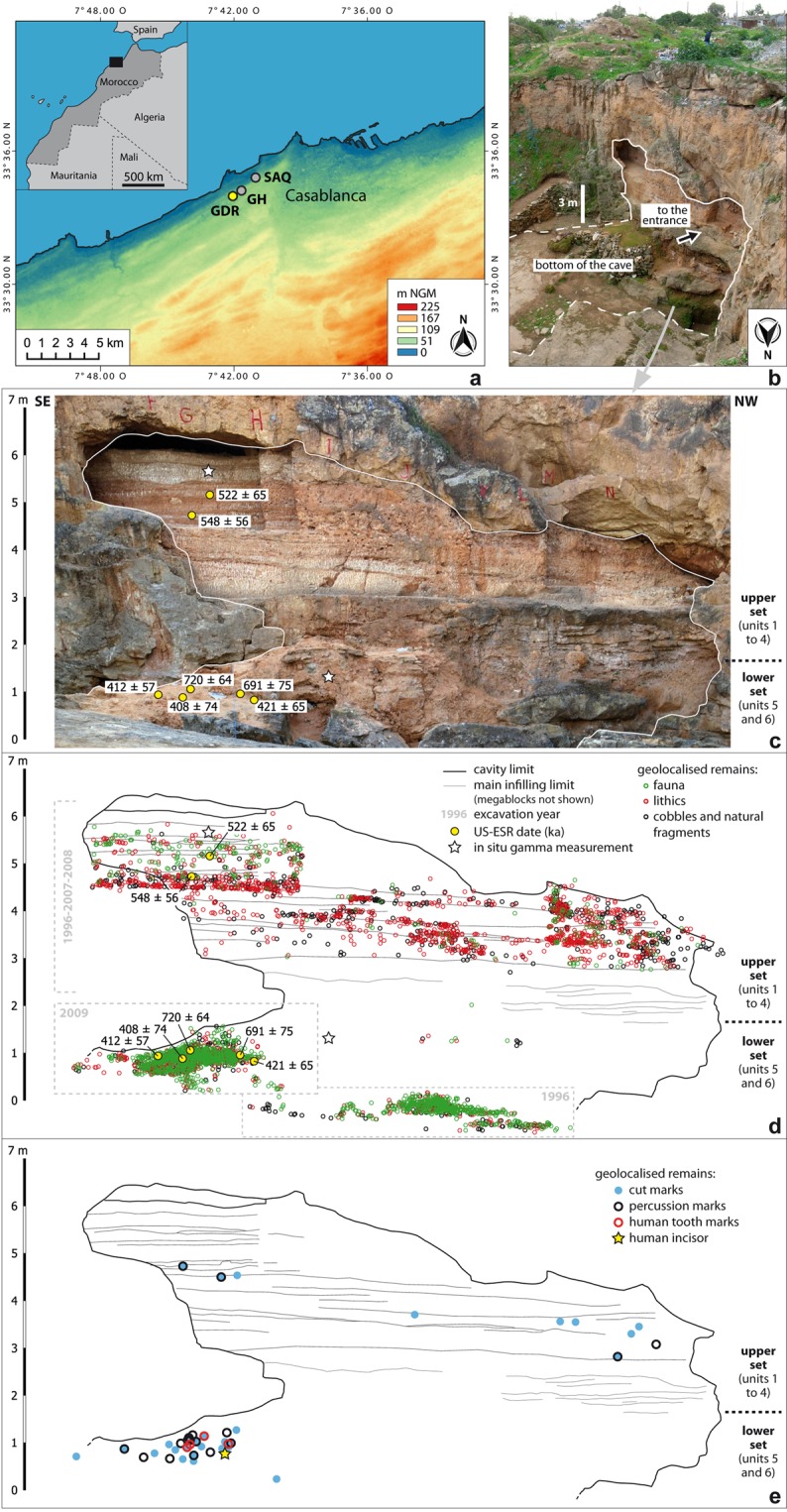
Location, US-ESR dates and vertical distribution of the remains of the GDR site. (**a**) Location of the site on the Moroccan Atlantic coast. GDR: Grotte des Rhinoceros, GH: Grotte des Hominidés, SAQ: Sidi Abderrahmane Quarry (drawings by M. Rué). (**b**) Oblique view of the site and limits of the present-day cave (photo M. Rué). (**c**) Projection of the US-ESR dates (ka) on an orthophoto of the infilling (photo D. Lefèvre). (**d**) Projection of the US-ESR dates and all the coordinated remains (drawings by M. Rué). (**e**) Projection of the human-modified remains presented in this article (only those with XYZ coordinates) and of the hominin incisor (drawings by M. Rué).

The ‘Grotte des Rhinocéros’ was discovered in 1991, within the frame of the France-Morocco cooperative program Casablanca^50^, at Oulad Hamida 1 Quarry, an extension of the former Thomas III Quarry, located 1 km from the current Atlantic coast (Fig. 1a). At the moment of the discovery, the quarry was active and had already destroyed the back part of the cave; only the lower part of the stratigraphy was visible. Systematic excavations were performed in 1996 and pursued from 2005 to 2009. These excavations yielded a rich Acheulean lithic assemblage associated with a rich, diverse fauna in a well-established stratigraphic context (see Supplementary Information Fig. S2). It was the first Moroccan Acheulean site dated by ESR^51,52^ and was chosen as the type-locality for the Second Regional Acheulean (SRA)^53^ (see Supplementary Information). The cave belongs to an early Middle Pleistocene shoreline of the Oulad Hamida Formation. The chronostratigraphic position within the succession of Pleistocene paleoshorelines at Casablanca indicates an age older than MIS 15 for its formation^53,54^ (and see the SI references 5 to 7). It is a large marine cavity of unknown total length, whose minimum dimensions at the bottom intersected by the quarry face are about 7 m high and 12 m wide (Fig. 1b). The cavity was filled by a continental 7 m-thick stratified accumulation of calcareous sandy materials divided into two main lithostratigraphic sets: the upper set (units 1 to 4) and the lower set (units 5 to 6) (see Supplementary Information Fig. S1). The deposition of fine sediment by low energy processes (aeolian and runoff) is responsible for the good preservation of the archaeological assemblages composed of bones and lithics (Fig. 1c–e). Facies and architecture of the deposits rule out the hypothesis of a pitfall accumulation.

## Results

### Dating

New combined ESR with U-series dating of seven bovid and rhinocerotid teeth from both lower and upper sets give ages of 720–690 ka and 548–522 ka respectively (see Supplementary Information; Tables S1 and S2; Figs. 1c and S1a). The homogeneity of the external dose rate allowed the calculation of an isochron age of 696 ka in good agreement with the oldest ages (unit 5). Three bovid teeth coming from this unit exhibit high uranium content in enamel probably limiting the creation of electron traps and leading to an underestimation of the DE (Equivalent Dose) and of the ages ranging between 408 and 421 ka (Fig. S3). This suggests that teeth have undergone a complex geochemical history such as uranium uptake in the enamel after the deposition while the dentine underwent a slight leaching probably during a humid period contemporaneous with a highstand sea-level (MIS17; see Supplementary Information). Two other teeth localized in unit 2 yield ages of 548–522 ka in agreement with the stratigraphic sequence. Our results are consistent with those obtained by Rhodes *et al*.^52^ on fossils from the lower set.

### Faunal assemblage

The faunal assemblage lacks *Homotherium* and *Metridiochoerus* and looks younger than that of Tighennif in Algeria, a site that may be about 1 Ma^55^, but the GDR rodents are closer to those of Tighennif than to those of Jebel Irhoud (Morocco), recently dated to c. 315 ka^49^. Additionally, the large number of extinct forms (see Supplementary Information), and the similarities of some of them with early Pleistocene taxa point to an early stage of the evolution of Middle Pleistocene assemblages, at certainly more than 0.5 Ma, which is consistent with the combined US-ESR dating.

Bovids are largely dominant among herbivores throughout the sequence, especially gazelles and Alcelaphini, mainly represented by cf. *Parmularius*. Gazelles and Alcelaphini are equally distributed among all age classes (Tables S3 and S4). Small-(S1–2) and medium-sized (S3) bovids have the same skeletal profiles, i.e. the predominance of cranial remains (without isolated teeth) and upper limb elements. The white rhinoceros *Ceratotherium mauritanicum* comes right after with 16.1% and 19.1% respectively of the NISPt in the lower and the upper sets. Both young and old individuals dominate. The rhinoceros skeletal profile is slightly different from those of bovids, with the decrease of limb extremities whereas axial elements are more common (Fig. S4).

Many other large mammals are present but in low quantity, such as zebras, warthogs, camels, an elephant and the Primate *Theropithecus oswaldi*.

Carnivores are more common in the upper set with a rate of 19.1% of the NISPt against only 7.6% in the lower set. In both series, the jackal *Lupulella mohibi* dominates, representing about half of the carnivores. It is followed by hyenids, large felids, ursids and some small carnivores. A few coprolites have been recorded in the two assemblages.

**Figure 2:**
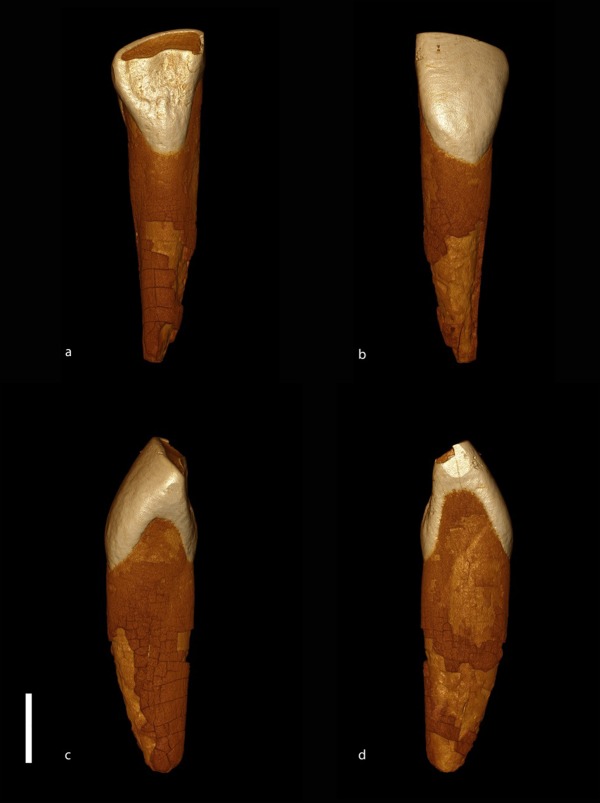
Surface model of the hominin incisor from unit 5 produced from CT data (by J.-J. Hublin). GDR09-8448 (lower set) is a well preserved left second incisor. It does not display any pathological conditions. The crown is worn and a substantial surface of dentine is exposed. Still, a large proportion of enamel is preserved above the cervix. Considering the rapid wearing of incisors in most Middle Pleistocene hominins, this tooth likely belonged to a young adult. The lingual surface of the crown displays a moderate shoveling, with well-developed mesial and distal ridges. The two ridges join a moderately expressed lingual cingulum out of which emerges a basal tubercle centrally placed. The mesial face is much wider than the distal face and both display well developed interproximal attrition facets. The buccal surface of the crown is supero-inferiorly convex, prolonging the profile of the buccal side of the root. The mesio-distal diameter of the crown (6.8 mm) is well within the variation observed among Middle Pleistocene European and African archaic hominins^119^, but the bucco-lingual diameter of 6.7 mm is at the lower limit observed in these groups^119^. The root has an ovoid section. Its apex was broken post-mortem and the preserved portion is 14.8 mm long from the lingual cervix.

Finally, the unit 5 also yielded a lower second incisor of archaic hominin (Fig. 2).

### Taphonomic context

The limited differential bone preservation and the well-preserved bone surfaces indicate a very good state of assemblage preservation (Fig. S5). The percentages of surface illegibility are small (6–12%), but there are differences between levels with more badly preserved surfaces being more common in the upper set (Tables S5 and S6) (Fig. S5). The good bone preservation is confirmed by the low bone destruction values (Tables S3 and S4). Most of the articular portions of long bones have been spared. Indeed, the epiphysis/diaphysis ratio is high for the two series, showing a good preservation of the spongious parts of the long bones.

**Figure 3:**
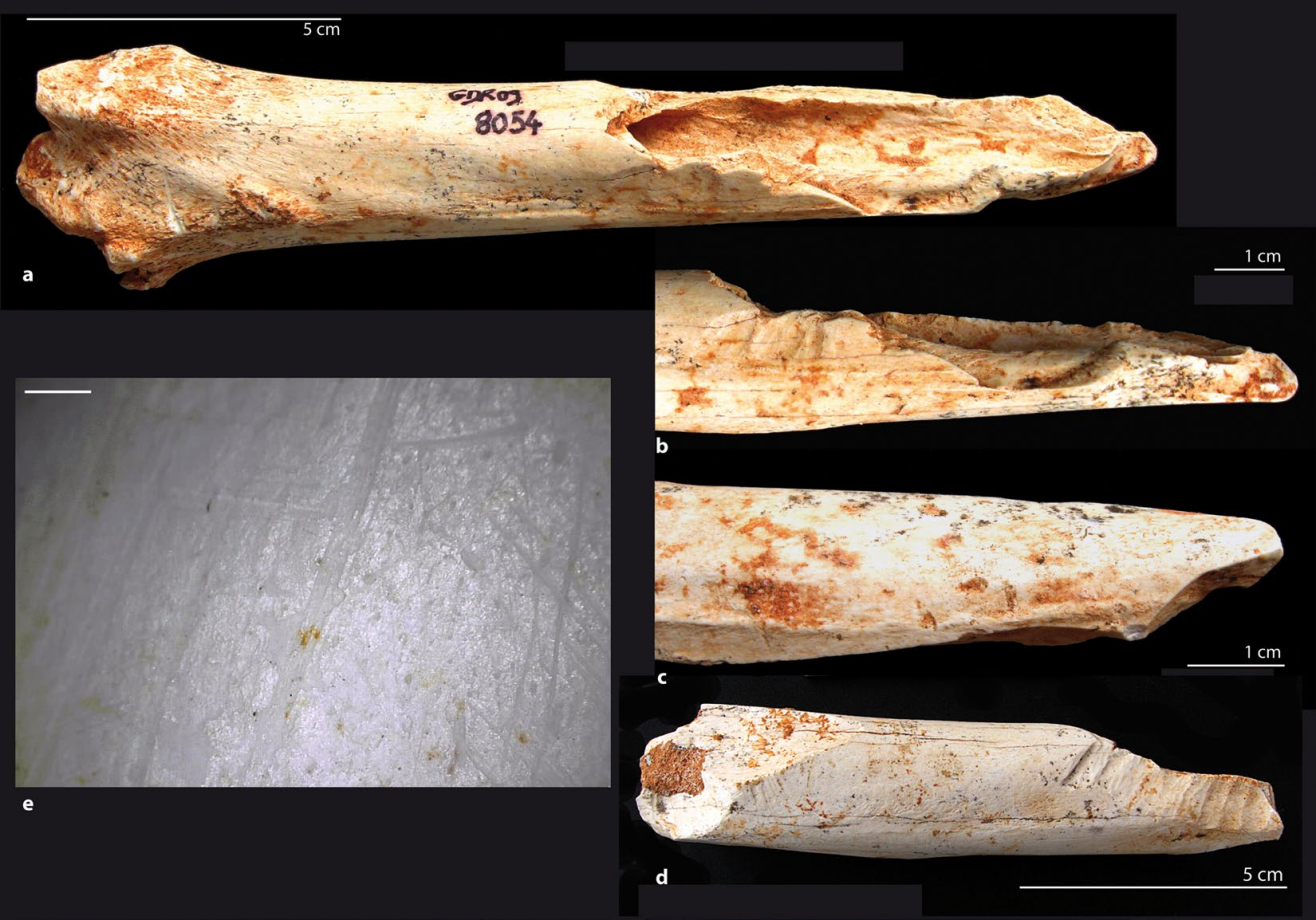
Non-anthropogenic bone surface modifications (lower set). Photos and digital microscope images by C. Daujeard. (**a–c**) Green bone breakage on a distal left tibia of an Alcelaphini (GDR09-8054). Notice the associated porcupine and carnivore tooth marks on the extremity of the bone fragment. (**d**) Shaft fragment of a bovid tibia with porcupine tooth marks (GDR09-7771). (**e**) Micro-abrasion striations on a distal shaft fragment of an alcelaphin femur (GDR09-8101).

The carnivore tooth-marks (pits, scores, punctures, notches) and ingested remains are more numerous at the base of the sequence and all species have been impacted, including jackals and hyenas (Tables S7 and S8; Fig. 3a–c). About 20% of the NISP of the bone remains in the lower layers and 15% in the upper ones present carnivore tooth-marks (Fig. 3a,c); some of them have been ingested. Some coprolites and ingested bones indicate the involvement of large carnivores, but the majority of the tooth mark and coprolite measurements show that they were left by the jackal. Carnivore tooth marks are equally distributed along the herbivore long bone elements, present on the median shaft (22.7%), shaft ends (21.9%) and articular portions (28.6%).

Porcupine tooth marks are also present in greater quantity in the lower set, with about 4% of the remains, against only 0.2% in the upper part (Tables S7 and S8; Fig. 3b,d). Most of the rodent marks are parallel scores and striations located on the edges of the bones, fracture angles or salient parts (Fig. 3d).

Numerous abrasion striations are present in the lower part of the sequence with 7% of the total readable bone elements in this set while, by contrast, they are almost absent in the upper set with only one bone striated (Tables S7 and S8). The abrasion process did not cause other damage than random striations (Fig. 3e). Polishing (smooth edges) remains rare and equivalent in the two series (1–2%). Linear abrasion marks are dense networks of very fine, superficial and overlapping long and straight striations, randomly located and oriented (Fig. 3e). These traces result from the mechanical friction of the bone surfaces against the sediment grains or lithic artefacts by trampling or limited sediment movements.

Root marking, calcite encrustations and desquamation are stronger in the upper layers, partly covering some surfaces. Inversely, black colorations (manganese oxides) and cracking are more frequent in the lower set (Tables S5 and S6). Nevertheless, the assemblages appear to be well preserved and only slightly affected by post-depositional bone surface alterations (weathering does not exceed stage 2 all over the sequence).

Long bone fragmentation is intensive, therefore identification indices are moderate. Despite the presence of a few whole long bones (3–5%), we observe the same low bone completeness indices for the two series (Fig. S5). The cause of this intensive fragmentation is mainly green bone breakage, especially for ungulate long bones with 81.2% and 65.8% respectively of broken bones in the lower and the upper sets (Tables S5 and S6). Post-depositional dry bone fractures are also common. Green and dry bone breakage are much more common in the lower set (Fig. S5a). Regarding the size categories for the whole bone assemblages (including sieving remains), more than 60% of the remains measure between 0 and 25 mm (Fig. S5b). In the lower set, ungulate long bones show good shaft length and circumference preservation, with L1 and L2 categories nearly equivalent. Some shaft cylinders are also present (Fig. S5c).

### Human induced marks

**Figure 4:**
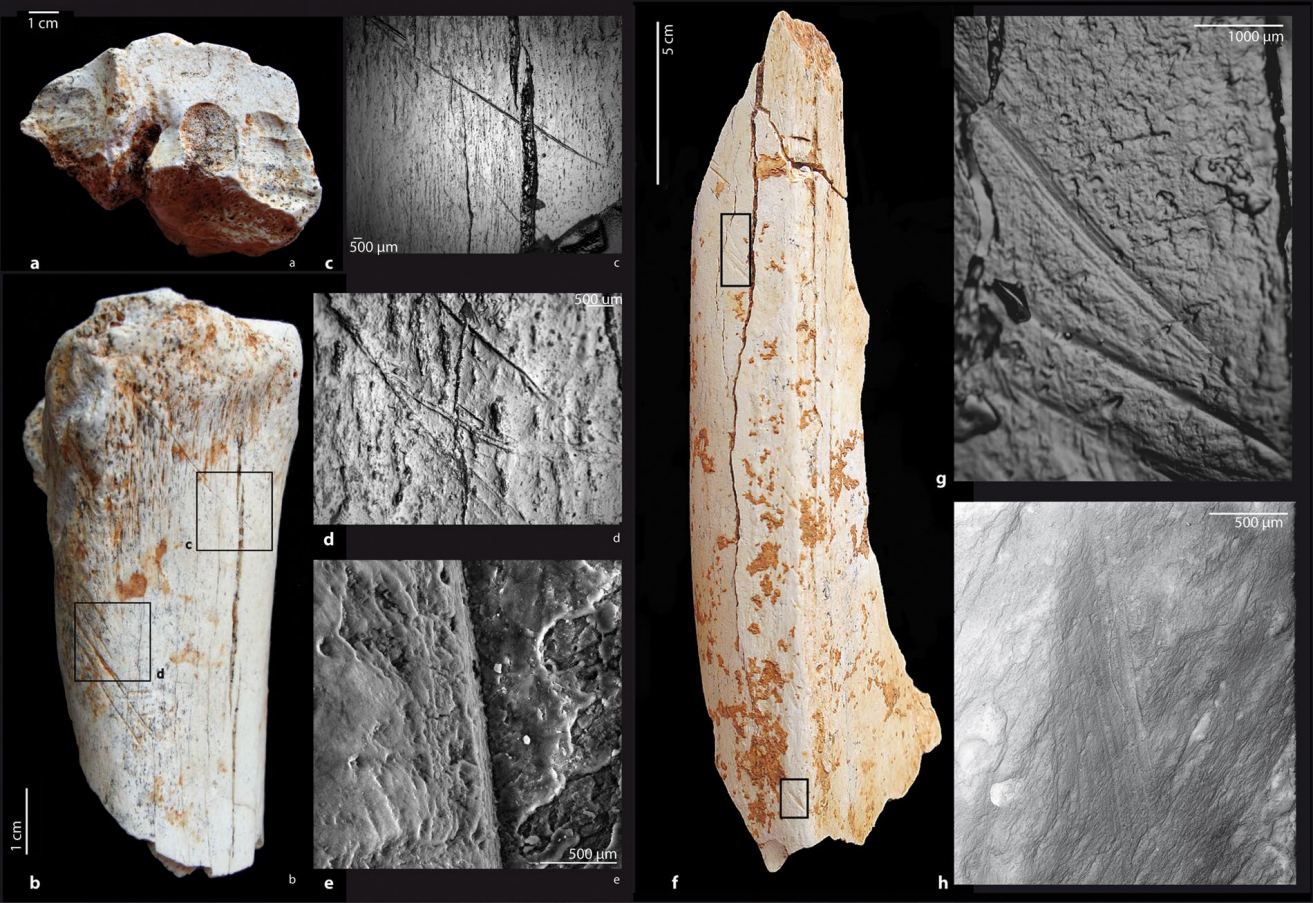
Cut marks on herbivore remains (lower set). (**a–e**) On a metacarpal proximal shaft fragment of an Alcelaphini (S3, cf. wildebeest) (GDR09-7159): (**a**) proximal view with some porcupine teeth marks and (**b**) medial side with a dozen long and parallel incisions. We observe clear shifts in the trajectory of some striations crossed by post-depositional cracks (**c**). Some of which have fork-shaped ends (**d**). Notice the V-shaped linear groove with internal striae for some of them (**e**). (**f–h**) On a radius-ulna medial shaft fragment of a zebra (*Equus mauritanicus*) (GDR09-7070): (**f**) view of the lateral side of the medial shaft with several dozen short and parallel incisions. Most of them have internal microstriations (**g**,**h**). All of the butchery marks (**a–h**) may be due to de-fleshing and/or disarticulation (images a,b, and f by C. Daujeard; Stereo microscope images c,d and g by G. Merceron and SEM images e and h by S. Pont).

**Figure 5:**
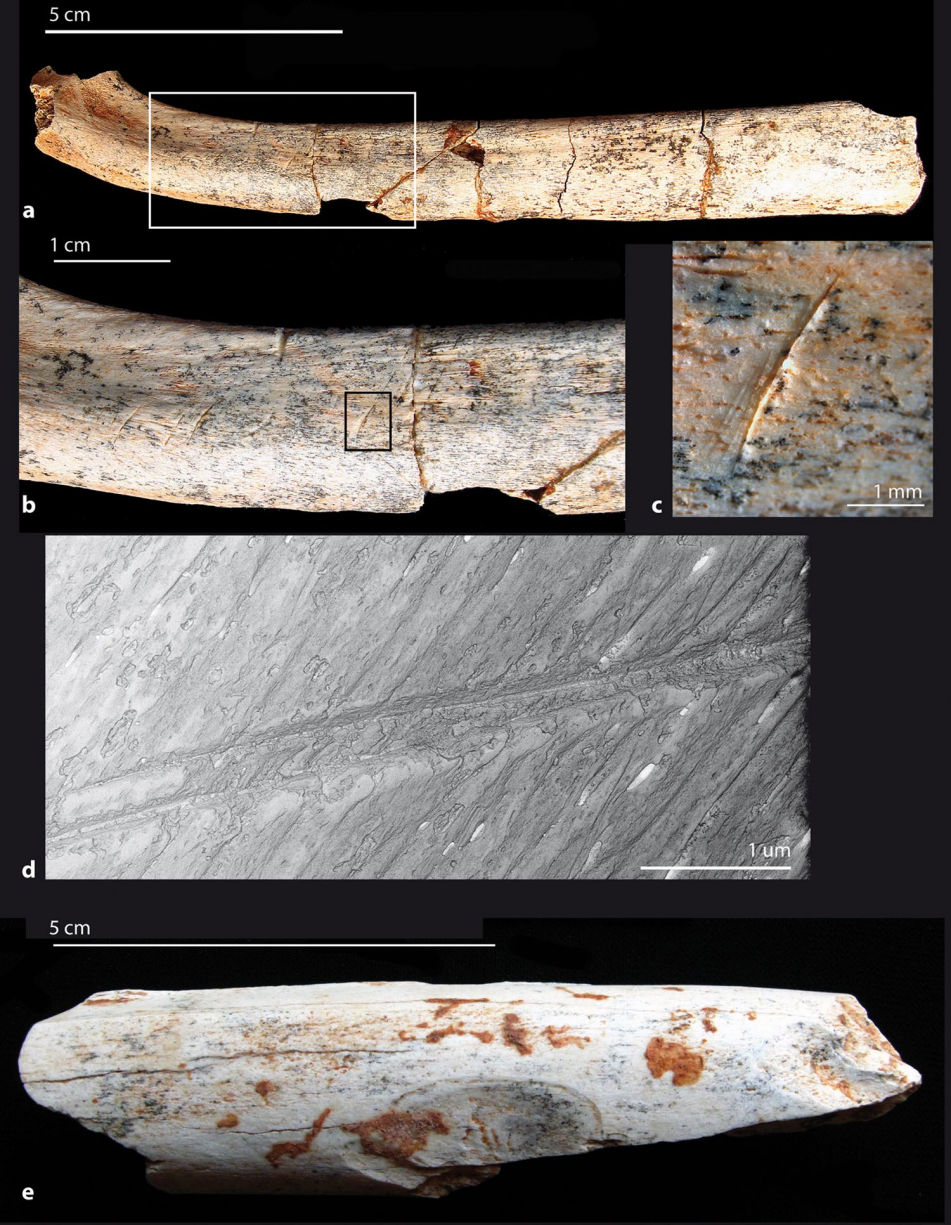
Cut and percussion marks on herbivore remains (lower set). (**a**,**b**) View of a dozen short and parallel incisions present on the proximal part of the external side of an alcelaphin rib (GDR09-6841). (**c**,**d**) Notice the shoulder effects and the V-shaped cross sections of some of the cut marks. These cut marks are interpreted as de-fleshing. (**e**) Percussion notch on a tibia shaft fragment of an indeterminate bovid S3 (GDR09-7438) (images a, b, c and e by C. Daujeard, c is a Stereo microscope image; SEM image d by S. Pont).

Beside these natural alterations on faunal remains, a number of bones display conclusive evidence of human-made marks (Figs. 4 and 5; see Methods section).

A total of 37 cut-marked specimens have been observed at GDR: 28 in the lower set and 9 in the upper one, which represent about 1% of the total readable bone elements for the two series (n = 2659 and 976 respectively; see Methods) and 3.8% and 5.5% of the identified bone remains (n = 531and 127) (Tables S7 and S8). Most of the marks are V-shaped incisions and display the same orientation within clusters, but are of various lengths, widths and depths. They occasionally present microstriations, shoulder effects, and fork-shaped ends (Figs. 4 and 5). Those marks are often isolated and circumscribed to muscle insertion reliefs. Other types of marks – superficial, parallel and more diffuse – are interpreted as scraping marks.

Various bovids (gazelles, wildebeest and *Parmularius*), zebras and unidentified bones display cut marks as well. They include long bones, ribs, vertebrae and pelvis, but only one short bone, a bovid calcaneum shows cut marks resulting from tarsus disarticulation. No clear butchery striations have been identified for the rhinoceros, as only three specimens (two ribs and one scapula) bear possible butchery marks unfortunately associated with trampling, carnivore and rodent modifications. Cut marks are unequally distributed along the long bone portions, with 12.5% for midshaft portions, 8.4% for shaft ends and 3.4% for epiphyses. For the two series, we have an equivalent number of cut-marked upper and intermediate long bones, respectively five and eight. The metapodials are slightly fewer with three cut-marked elements.

Percussion marks (flakes, notches or pits and grooves with microstriations) are rare, being present on less than 1% of the total readable bone elements and 4% of them being associated with green bone fractures (Fig. 5e); these percentages are lower than carnivore tooth marks (Tables S7 and S8). This suggests the greater involvement of carnivores in the breakage process. Most (about 75%) of the cut-marked specimens show green bone fractures, some of them associated with percussion marks, testifying to marrow recovering by hominins. Almost all of the percussion marks occur on zebra or bovid long bone shafts.

**Figure 6:**
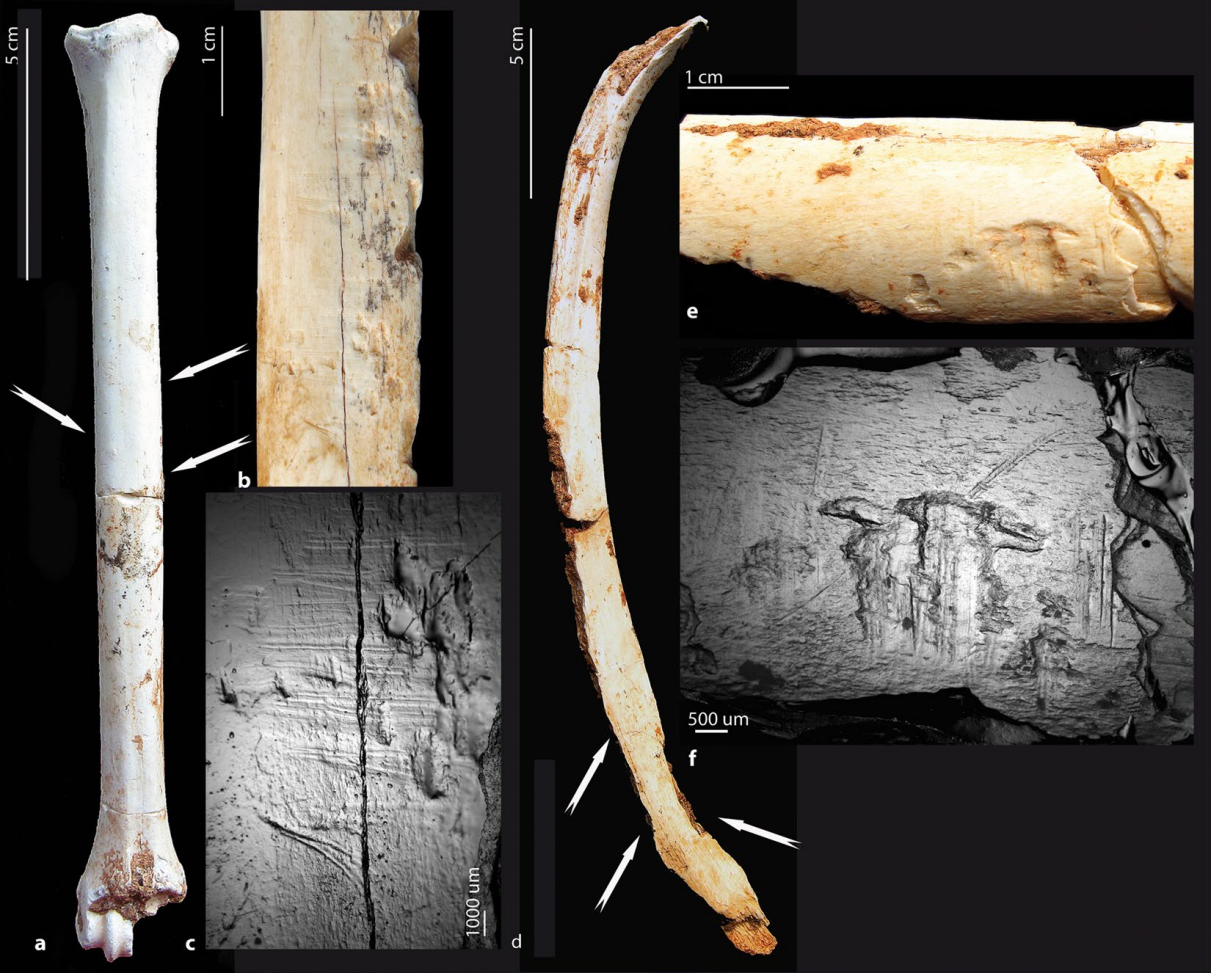
Human tooth marks on herbivore remains. (**a–c**) On a shaft fragment of a gazelle metatarsal (GDR09-7293, lower units). (**d–f**) On a shaft fragment of an alcelaphin rib (GDR0-7673, lower units). Notice that some human tooth marks are associated with anterior butchery marks (**f**) (images a,b,d and e by C. Daujeard; Stereo microscope images c and f by G. Merceron).

Some peculiar marks have been observed on four bone elements coming from the lower lithostratigraphic set. We identified them as human tooth marks (Fig. 6). They are shallow linear marks, crescent-shaped pits with internal and external scratches, triangular puncture marks and peeling on the surface. They are not found isolated, but clustered on the same element: the shaft portions of an alcelaphin rib and a gazelle metatarsal, the ilium of a bovid and a bone fragment. The extremity of the rib shows a green bone fracture with classic peeling associated with scores and butchery striations.

Below, we list the entire cut marked specimen by taxa, adding a complete description of the butchery marks and interpretations (Table S9).

#### The lower stratigraphic set

*Equus*: Cut marks on the zebra are documented on two mid-shaft fragments of a radio-ulna and a femur. About 40 striations are present on the lateral side of the radio-ulna shaft, along the fusion zone (Fig. 4f–h). They are short oblique and parallel incisions of variable depth and breadth. Some of them present shoulder effects, fork-shaped ends and microstriations resulting from defleshing. Six cut marks are present on the femur, on the internal face of the greater trochanter. They are thin and long incisions, parallel to the long axis of the bone, and also indicate the defleshing of the meaty part of the bone.

*Gazella*: Seven cut marked gazelle bones were observed. They are shaft fragments of a femur, a humerus, a metatarsal and an ulna. Long and oblique striations resulted from the defleshing of the meaty parts of the proximal segments of the limb (femur and humerus). Besides, some shorter cut marks occur on the lateral epicondyle and above the olecranon fossa of the humerus and are due to the disarticulation of the humerus-ulna joint. Marks on the metatarsal along the posterior gutter are interpreted as a skinning process. The ulna shows three long- and medium-sized oblique marks on its medial shaft, possibly due to defleshing. Two fragments of pelvis (ilium) and one spinous process of a thoracic vertebra also present butchery striations due to defleshing.

*Parmularius*: Six shaft fragments of long bones of *Parmularius* (one humerus, one radius, one femur and three tibias) show cut marks. Two rib fragments present striations on the external side (Fig. 5a–d). All of these elements present incisions resulting from the defleshing and only two, the femur and the radius, also have scraping marks, possibly related to periosteum removal. Most of the marks have microstriations, shoulder effects and fork-shaped ends. One of the two ribs shows human tooth marks and percussion impacts superimposed onto the cut marks (Fig. 6d–f).

*Connochaetes*: One medio-proximal fragment of a wildebeest metacarpal shows two clusters of long- and medium-sized, V-shaped, oblique incisions located on the anterior and medial sides of the proximal shaft (Fig. 4b–e). They present microstriations, shoulder effects and fork-shaped ends. They are interpreted as resulting from the defleshing and disarticulation of the carpo-metacarpal joint. On this specimen, we observed clear shifts in the trajectory of some striations crossed by post-depositional cracks, proving that the striations occurred prior to the cracks (Fig. 4c).

*Unidentified remains*: Ten cut marked specimens are non-identified, belonging to fragments of long bones, ribs and short articular bones. Three of them are indeterminate bovids of S2–3 (tibia medial shaft fragment and long bone unidentified elements). One is a calcaneum fragment of an indeterminate bovid of S3–4. Only one cluster of cut marks on the tibia corresponds to scraping marks. Otherwise, all marks are incisions of various dimensions and various depths, mostly parallel to each other and oblique in relation to the long axis of the bones.

#### The upper stratigraphic set

*Equus*: One medial shaft fragment of a zebra metapodial presents five cut marks on its posterior side. Those marks are short- and medium sized oblique, V-shaped striations, parallel or not. The process may be defleshing or skinning.

*Gazella*: Two fragments of pelvis and radius of gazelles show cut marks. Two clusters of striations are located on the anterior muscle insertion surface of the ilium. Marks are short, oblique and parallel or not. They are interpreted as the disarticulation of the femur-pelvis joint. The radius has three clusters of incisions and scraping marks on the medial shaft portion, related to defleshing and periosteum removal.

*Parmularius*: Three fragments of ribs of a size 2–3 bovid (cf. *Parmularius*) present cut marks. Incisions and scraping marks were recorded on the external sides of the ribs and medial shaft of the long bone. Most of the marks have microstriations and some have shoulder effects and fork-shaped ends. Defleshing and scraping are the two processes recognized.

*Unidentified remains*: Two indeterminate fragments and one fragment of an indeterminate long bone of a S2–3 bovid present cut marks. They are oblique and clustered incisions with fork-shaped ends and scraping marks.

## Discussion

The higher proportion of cracked and of biologically modified bones (by porcupines, carnivores and humans) in the lower part of the sequence suggests a longer exposure for these remains. Likewise, the natural abrasion striations occur only in the lower part, probably because of a longer exposure to animal and human trampling. No marks of carnivores and humans are found superimposed or present together, giving no indication of the order of events and demonstrating distinct events of butchery and carnivore activity^56^. The very low rates of cut (~1%) and percussion (~0.5%) marked specimens in relation to the carnivore tooth marks (~4–8%) suggest that hominins were not the main agent of accumulation (Tables S7 and S8). The number of carnivore tooth marks and their even distribution among herbivore long bone ends and midshaft portions indicate that they were an important agent of accumulation, or at least of bone surface modification and bone-breaking. Nevertheless, the presence of some whole bone elements and the relatively high ratio of epiphysis/diaphysis (0.4–0.6; Tables S3 to S6) do not support a strong contribution of the hyenids to the taphocenosis, nor the exclusive use of the cave as a den. The dimensions of tooth marks and coprolites indicate that different sizes of carnivores are responsible for the modifications and that the jackal is the most important destructive agent.

By contrast, the role of hominins was reduced with some limited butchery evidence (cut and percussion marks). Yet, the greatest frequencies of cut marks on the midshafts indicate the defleshing of the meaty long bone portions. The ratios of cut-marked meaty-bone mid-shaft portions to cut-marked metapodial mid-shaft portions, which is 19/3 for the lower set and 3/1 for the upper set, actually suggest that hominins had primary access to at least some carcasses^19^. As well, the meaty upper and intermediate limb bones which have a nearly equivalent number of cut marked elements, respectively 5 and 7 in the lower set while only two lower limb bone elements (metapodials) have been cut marked, also point to primary access^19^.

The age and skeletal profiles for S1 to S3 bovids are in favour of predation activities, while larger ungulates such as zebras, S4 bovids and rhinoceroses seem to have been scavenged instead. Part of the faunal remains have been accumulated by carnivores and to a lesser extent by human transport, but the moderate bone destruction points also to a non-biotic origin.

The presence of numerous rhinoceros carcasses in the cave is intriguing. Even if we take into account the thickness of the skin and flesh of this very large mammal and the illegibility of some bone surfaces, we note a total absence of human-made marks and by contrast the presence of a few carnivore tooth marks. The presence of more than a dozen skulls (some broken and with disarticulated jaws), along with ribs, limb parts, pelvises and scapulas, would suggest the carnivore and/or human exploitation of animals *in situ*, but this issue requires further investigation.

At GDR, the spatial and temporal organization differs from that of the earlier Acheulean open-air sites in Eastern^8,9,12,13,19,20,32^ and Northern Africa^43–45^. Indeed, the oldest evidence of butchery in Africa occurred in open-air sites, where hominins had early access to carcasses in kill- or active scavenging-sites, occasionally visited by carnivores^57–62^. Some “central-place” early Acheulean sites have been identified in Africa, often related to megafauna exploitation dating to less than 1.7 Ma^12,20^, but still in open-air contexts. In most of these sites, it is difficult to draw conclusions regarding site function, hominid access to the carcasses, meat consumption, transport of carcass portions, and interactions between humans and carnivores^6,22,63^. Moreover, post-depositional agents often affect archaeological assemblages and limit the interpretations so that *in situ* animal consumption is more often taken for granted than proved by detailed taphonomic analyses. At GDR, hominins carried out subsistence activities in the cave, at least on some small- to large- sized herbivores, mostly on gazelles, zebras and Alcelaphini, and thus configured caves as “central places” in the landscape. The abundant lithic industry associated with fauna remains in both archeological units with huge flaking activity and LCT’s (bifaces and cleavers) shaping give weight to this interpretation. Finally, according to experimentations^64^, fork-shaped marks are among the variables capable of discerning cut marks produced with lithic handaxes. This morphology is frequent here, and possibly related to the abundance of LCT’s in the lithic assemblage.

Occupation of caves as persistent dwellings is an important behavioral marker in human evolution. It provides many advantages such as protection against environmental events, fire maintenance, as well as the development of a structured space for prolonged secondary butchery and feeding^65–68^. Here, the association of cut-marked remains and stone tools in a cave context within a taphonomically interpretable assemblage demonstrates that this important step occurred in Northern Africa at about 700 ka. Indeed, GDR is one of the few early Middle Pleistocene sites – and for now the only one in Africa – in which human animal consumption is directly associated with Acheulean stone tools manufacture in a cave^6,32,34,43^.

## Methods

### Material for taphonomic analysis

The taphonomic analysis concerns all the faunal remains of large mammals coming from the 2007 to 2009 excavations, including those recovered by sieving. A preliminary taphonomic analysis was recently published for the faunal assemblage from earlier excavations^69^. Length, breadth, thickness, breakage and bone surface modifications were recorded for all 3D plotted elements and for all identified ones (non-plotted included). Indeterminate sieving fragments were only used for counts, fragmentation studies (type and size classes) and biological agents (carnivores, rodents or humans). We report here the total number of remains (NR), the number of identified specimens taxonomically and anatomically (NISPt and NISPa) as well as the minimum number of individuals (MNI). The sample has been divided into two main assemblages coming from the upper set and the lower set. The first one contains a total of 1097 remains and the second one 2873 remains (Tables S3 and S4). For indeterminate remains we defined the type of specimen (compact; spongy; tooth; long or short bones) and established size and weight categories (Table S10).

### Carcass transport strategies

In order to discuss transport strategies and/or differential bone preservation, we used the percentage of Minimal Animal Unit^70^ (MAU) and the bone bulk density^71,72^. Carcasses were divided into seven main anatomical parts: head (skull, mandibles, isolated teeth excepted); axial elements (vertebrae, ribs, sternum); forequarters (scapula, humerus, radio-ulna); forefoot (carpal, metacarpal); hindquarters (pelvis, sacrum, femur, tibia, patella); hindfoot (tarsal, metatarsal); and indeterminate foot (phalanges, sesamoids, metapodials). Ontogenic age-at-death is based on dental eruption and replacement patterns and then on tooth wear. We used the method of Munro *et al*.^73^ to assess the age of gazelles, Bunn and Pickering^74^ for the other bovids, and Hitchins^75^ and Tong^76^ for rhinos.

### Breakage processes

The identification of the type of breakage (green, dry or recent bone fractures) was made based on the fracture colour, shape, feature and angle^77^. The identification of the breaking agent is based on the morphology of the percussion marks and associated bone surface modifications^78,79^. The recorded percussion marks are medullary or cortical percussion notches (negative flake scars); impact flakes (positive flake scars) or percussion pits^78,80^. The shaft fragments were differentiated by size and circumference classes^77^.

### Bone surface modifications analysis

In order to distinguish depositional and post-depositional damages, all bone surfaces were observed using the naked eye and a 10x hand lens under high incident light^81^, with some further assessed using a Digital microscope (Dino-Lite AD7013MZT; 20x to 200x). We also made a selection of key samples for higher resolution and magnification images with a Leica S8 APO stereomicroscope (10x to 80×), a scanning electron microscope (SEM) and a 3D Digital Microscope (Hirox). Those key samples were selected according to their type and intensity of bone modifications, for example we prioritized the elements bearing the most visible and clear bone modifications (human, animal or trampling marks).

The number of bone remains analyzed for the bone surface modifications (NRtaph) is 386 for the upper set and 986 for the lower set (Tables S3 and S4). However, to quantify cut marks, and other biotic modifications, we took into account the sieved remains. Only specimens with visible bone surfaces were taken into account in the percentages. We excluded the bone specimens having a completely illegible bone surface (=0) due to post-depositional alterations. Indeed, we categorized the bone elements of our series accordingly: 0 = unreadable bone surfaces; 1/3 of readability; 2/3 of readability and 1 = entirely readable. The total number of observed (readable) elements are 976 and 2659 for the upper and lower sets respectively (Tables S7 and S8).

The type and the location of modifications were recorded. We distinguish between those made by rodents, carnivores or hominins, as well as climatic and edaphic modifications (including cracking, desquamation, polish, concretion, root etchings, natural abrasion, chemical corrosion and oxides colorations), on the basis of the criteria defined in the literature^81–88^.

#### Carnivore and rodent traces

Carnivore marks were classified as follows: pits, punctures, scores, notches or corrosion by gastric acids and their locations on long bones were recorded^86,87,89^. We took carnivore tooth mark measurements (maximal length and breadth) into account, as well as tissue location (cancellous bone or articular portions; cortical or median diaphysis; thin cortical bone or diaphysis extremity). While insufficient when taken alone, measuring the tooth marks (especially maximal dimensions) is the most accurate method for establishing the body size of the predator^90–92^. We also measured the coprolites (length, breadth and thickness). Traces left by rodent incisors are parallel, wide or narrow furrows, sometimes including internal micro-striations^85,93^.

#### Cut marks identification

Particular attention has been given to cut marks, observed under low (10x−40x) and high magnification (up to 80x with the stereomicroscope and to 200x and more with the Dinolite and SEM). We distinguished natural abrasion striations (i.e. trampling marks) from butchery marks using previous experimental and descriptive works^64,83,84,86,88,94,95^. We used the following variables to discriminate cut marks from natural abrasion: dimensions (length); width and shape of the cross-section (narrow and wide V-shape); depth (deep, moderate or superficial); internal microstriations; shoulder effect; flaking; trajectory (straight or sinuous linear grooves); isolated or grouped striations and relationship (parallel, non-parallel or overlapping); orientation with respect to the major axis of the bone (parallel, oblique or perpendicular); presence of fork-shaped marks or multiple grooves (in some case of cut marks made with lithic handaxes); frequency and location of the marks on the bone (rather muscle and tendons attachment areas for cut marks and more likely a random location for natural abrasions). Finally, because the morphology of trampling marks can mimic cut marks, even microscopically, only the combination of several of those criteria (morphology, quantity, context, location) allowed us to strongly identify evidence of butchery, i.e. a series of parallel and deep V-shaped grooves with straight and continuous internal microstriations located on some muscle attachment areas are more likely to be cut marks. The association of cut marks with natural abrasion striations is also taken into account in our identification process. Thus, in a contextual approach, we excluded from our counts all remains that displayed possible cut marks found in direct and clear association with natural striations. As stated above, we also excluded all bones with illegible surfaces. Consequently, our cut marked specimen frequency is certainly underestimated. Although Early Palaeolithic assemblages often contain a smaller number of cut marked bones because of the state of preservation and of the onset of carnivorous diet^96–98^, the frequency of cut marked specimens is also a strong and valuable diagnostic criteria to identify human consumption activity.

Once the types of cut-marks, scraping marks or incisions^99^, were recorded, their location and morphology were used to indicate the related butchering activity including evisceration; skinning; dismemberment; disarticulation; periosteum removal; cutting tendons and de-fleshing^22,86^. We reported the name of each muscle and tendon affected by the well-located cut marks. Lastly, to differentiate primary from secondary access to the carcass we used the distribution of cut marks both per bone section (ratio of cut marked diaphyses on cut-marked epiphyses) and per bone elements (ratio of cut-marked meaty-bone shafts on cut-marked metapodials shafts)^19^.

#### Deciphering human tooth marks

Human tooth marks may be shallow linear marks showing a crescent pit and internal scratches along the bottom of the groove, “double-arched shape” punctures on crenulated edges, triangular puncture marks, or peeling on the surface^86,100–105^. Shallow linear marks could be difficult to decipher with the naked eye and thus necessitate the need be seen at high magnification. Experiments have shown that those marks are usually produced by human incisors^103^. Thus, at GDR, we paid particular attention to this type of damage, even if its occurrence in sites where multiple accumulators interact is difficult to demonstrate.

### Dating methods

The combined ESR/U-series (US-ESR) model takes into account both ESR and U-series data including radioelement contents, isotopic ratios, equivalent dose and external gamma-dose rate allowing for the reconstruction of the uranium uptake history in each dental tissue using a specific U-uptake parameter (p-value)^106^. The model cannot account for uranium loss. A new model combining ESR and U-series data (called the Accelerating uptake model, AU-ESR) allows to analyse samples exhibiting slight uranium leaching^107^. The application of the combined approach has been used for dating the entire Middle Pleistocene on both human and animal teeth^108,109^.

Seven herbivorous teeth, unearthed from the 2007–2009 excavation campaigns, were analysed using the ESR/U-series method at the Geochronology Lab of the department of “Homme et Environnement”, Muséum National d’Histoire Naturelle, Paris. The samples come from two areas: A first zone has yielded 5 teeth (3 bovids and 2 rhinocerotids), corresponding to E15, D16 squares and to the stratigraphical unit 5 very close to the human tooth marks. A second area, excavated earlier, provided two teeth (one bovid and one rhino) in G19, G20 squares (Extended Data Fig. 3). The samples are in a good state of conservation and are embedded in a reddish sediment (Extended Data Fig. 2).

Enamel and dentine were separated mechanically and their radioisotope contents measured by U-series using alpha-ray spectrometry or ICPMS-MC according to standard methods^110,111^, and gamma-ray spectrometry^112^. No cement was observed in the selected teeth. Enamel was cleaned on both inner and outer sides to eliminate the effects of external alpha radiation. It was then ground, sieved at 100–200 µm fraction and split into 10 aliquots used for ESR measurements.

Nine of them were irradiated with a calibrated ^60^Co gamma-ray panoramic source (LABRA, CEA, Saclay, France) from 160 to 11700 Gy.

ESR measurements were performed in a Paris lab at room temperature on an EMX-6 Bruker spectrometer (X band, 9.82 GHz) with a microwave power of 1 mW and with a modulation amplitude of 0.1 mT. A scan range of 10 mT and a scan time of two minutes with a modulation frequency of 100 kHz were used for each spectrum. Each ESR measurement was repeated four times for each dose over different days.

ESR intensities were extracted using the Bruker WINEPR System software from the asymmetric ESR signal between the T1-B2 signal at g = 2.0018^113^. ESR intensities were fitted by an exponential plus linear function (EXPLIN) using Origin Pro8 software (OriginLab Corporation, Northampton, USA) to determine the equivalent dose (DE). Data were weighted by 1/I2. US-ESR age calculations were carried out with the ESR-DATA program^114^ which uses an alpha efficiency of 0.13 ± 0.02^115^ and Monte-Carlo beta attenuation factors^116^ based on the thickness of tooth enamel and after the removal of the outer layers. The age of some samples exhibiting weak uranium leaching were calculated using AU-ESR model^107^. The water content was estimated to be 7 ± 3% in the dentine and 12 ± 5% in the sediment (dry weight versus wet sediment in an oven at 35 °C). No water content was taken into account in the enamel. Gamma-ray spectroscopy measurements were performed using a high purity low-background Ge detector to determine specific activity of radioisotopes (U, Th, K) in the sediments including the samples. *In situ* measurements were performed using a γ-ray portable spectrometer Inspector 1000 Canberra with NaI detector. The dose rate was calculated according to Guérin *et al*.^117^. The effect of Ra and Rn loss in each tissue was determined by combining alpha-ray and gamma-ray measurements^118^.

## Supplementary information

Supplementary Information.

## References

[1] Smith GM, Ruebens K, Gaudzinski-Windheuser S, Steele TE (2019). Subsistence strategies throughout the African Middle Pleistocene: Faunal evidence for behavioral change and continuity across the Earlier to Middle Stone Age transition. J. Hum. Evol..

[2] Bahram, L. & Mitchell, P. First Africans: African Archaeology from the Earliest Tool Makers to Most Recent Foragers. (Cambridge, 2008).

[3] Chazan M (2008). Radiometric dating of the Earlier Stone Age sequence in Excavation I at Wonderwerk Cave, South Africa: preliminary results. J. Hum. Evol..

[4] Berna F (2012). Microstratigraphic evidence of *in situ* fire in the Acheulean strata of Wonderwerk Cave, Northern Cape province, South Africa. Proc. Natl. Acad. Sci..

[5] Brink J, Holt S, Horwitz LK (2016). The Oldowan and Early Acheulean Mammalian Fauna of Wonderwerk Cave (Northern Cape Province, South Africa). African Archaeol. Rev..

[6] Kuman, K. The earlier Stone Age in South Africa: site context and the influence of cave studies. In *Breathing life into fossils: Taphonomic studies in Honor of C.K. (Bob) Brain* (eds. Pickering, T. R., Schick, K. & Toth, N.) 181–198 (Stone Age Institute Press, 2007).

[7] Forrest FL (2018). Journal of Archaeological Science: Reports Zooarchaeological reconstruction of newly excavated Middle Pleistocene deposits from Elandsfontein, South Africa. J. Archaeol. Sci. Reports.

[8] Asfaw B (1992). The earliest Acheulean from Konso-Gardula. Nature.

[9] Lepre CJ (2011). An earlier origin for the Acheulian. Nature.

[10] Suwa, G., Nakaya, H. & Asfaw, B. The Konso Formation Paleontological Assemblages: Collecting and Documentation Methodologies. In *Konso-Gardula Research project* (eds. Suwa, G., Beyene, Y. & Asfaw, B.) 5–9 (University of Tokyo, 2014).

[11] Echassoux A (2012). Comportements de subsistance et modifications osseuses à l’aube de l’Acheuléen à Konso, Éthiopie. Anthropologie.

[12] Diez-Martín F (2015). The Origin of the Acheulean: The 1.7 Million-Year-Old Site of FLK West, Olduvai Gorge (Tanzania). Sci. Rep..

[13] Semaw, S. The Early Acheulean ~1.6–1.2 Ma from Gona, Ethiopia. In *The Emergence of the Acheulean in East Africa and Beyond* (eds. Gallotti, R. & Mussi, M.) 115–128 (Springer, 2018).

[14] Gallotti R (2013). An older origin for the Acheulean at Melka Kunture (Upper Awash, Ethiopia): Techno-economic behaviours at Garba IVD. J. Hum. Evol..

[15] Fiore, I. & Tagliacozzo, A. Taphonomic analysis of the bone remains from the Oldowan site of Garba IV. In *Studies on the Early Paleolithic Site of Melka Kunture, Ethiopia* (eds. Chavaillon, J. & Piperno, M.) 639–682 (Istituto italiano di preistoria e protostoria, Florence, 2004).

[16] Domínguez-Rodrigo, M., Serrallonga, J., Luque, L., Diez-Martín, F. & Bushozi, P. The archaeology of the Acheulean sites from South Escarpment. In *Peninj. A Research Project on the Archaeology of Human Origins* (1995–2005) (eds. Domínguez-Rodrigo, M., Alcalá, L. & Luque, L.) 205–226 (Oxbow, 2009).

[17] Domínguez-Rodrigo, M., Serrallonga, J., Luque, L., Diez-Martín, F. & Bushozi, P. The archaeology of the North Escarpment. In *Peninj. A Research Project on the Archaeology of Human Origins* (1995–2005) (eds. Domínguez-Rodrigo, M., Alcalá, L. & Luque, L.) 227–256 (Oxbow, 2009).

[18] Diez-Martín F (2014). Early Acheulean technology at Es2-Lepolosi (ancient MHS-Bayasi) in Peninj (Lake Natron, Tanzania). Quat. Int..

[19] Domínguez-Rodrigo M (2009). Unraveling hominin behavior at another anthropogenic site from Olduvai Gorge (Tanzania): new archaeological and taphonomic research at BK, Upper Bed II. J. Hum. Evol..

[20] Domínguez-Rodrigo M (2014). On meat eating and human evolution: A taphonomic analysis of BK4b (Upper Bed II, Olduvai Gorge, Tanzania), and its bearing on hominin megafaunal consumption. Quat. Int..

[21] Braun DR (2010). Early hominin diet included diverse terrestrial and aquatic animals 1.95 Ma in East Turkana, Kenya. Proc. Natl. Acad. Sci..

[22] Pobiner BL, Rogers MJ, Monahan CM, Harris JWK (2008). New evidence for hominin carcass processing strategies at 1.5 Ma, Koobi Fora, Kenya. J. Hum. Evol..

[23] Merritt SR (2017). Investigating hominin carnivory in the Okote Member of Koobi Fora, Kenya with an actualistic model of carcass consumption and traces of butchery on the elbow. J. Hum. Evol..

[24] Hay, R. L. *Geology of the Olduvai Gorge*. (University of California Press, 1976).

[25] Isaac, G. L. & Isaac, B. *Koobi Fora Research Project, Plio-Pleistocene Archaeology*. (Clarendon Press, 1997).

[26] Roche H (2003). Les sites archéologiques plio-pléistocènes de la formation de Nachukui, Ouest-Turkana, Kenya: bilan synthétique 1997–2001. Comptes Rendus - Palevol.

[27] Quade J (2008). The Geology of Gona, Afar, Ethiopia. Geol. Soc. Am. Bull..

[28] de la Torre I (2011). The Early Stone Age lithic assemblages of Gadeb (Ethiopia) and the developed Oldowan/early Acheulean in East Africa. J. Hum. Evol..

[29] Beyene Y (2013). The characteristics and chronology of the earliest Acheulean at Konso, Ethiopia. Proc. Natl. Acad. Sci..

[30] Gallotti, R. & Mussi, M. The Emergence of the Acheulean in East Africa: Historical Perspectives and Current Issues. In *The Emergence of the Acheulean in East Africa and Beyond* (eds. Gallotti, R. & Mussi, M.) 1–12 (Springer, 2018).

[31] Delagnes A (2006). Interpreting pachyderm single carcass sites in the African Lower and Early Middle Pleistocene record: A multidisciplinary approach to the site of Nadung’a 4 (Kenya). J. Anthropol. Archaeol..

[32] Altamura F (2018). Archaeology and ichnology at Gombore II-2, Melka Kunture, Ethiopia: everyday life of a mixed-age hominin group 700,000 years ago. Sci. Rep..

[33] Altamura, F., Gaudzinski-windheuser, S., Melis, R. T. & Mussi, M. Reassessing Hominin Skills at an Early Middle Pleistocene Hippo Butchery Site: Gombore II-2 (Melka Kunture, Upper Awash valley, Ethiopia). *J. Paleolit. Archaeol*, 10.1007/s41982-019-00046-0 (2019).

[34] Pante MC (2013). The larger mammal fossil assemblage from JK2, Bed III, Olduvai Gorge, Tanzania: Implications for the feeding behavior of Homo erectus. J. Hum. Evol..

[35] Balout, L. *Préhistoire de l’Afrique du Nord. Essai de chronologie*. (Arts et métiers graphiques. Paris, 1955).

[36] Vaufrey, R. *Préhistoire de l’Afrique*. (Publication de l’Institut des Hautes Etudes de Tunis, 1955).

[37] Biberson, P. *Le Paléolithique inférieur du Maroc atlantique*. (Publications du Service des Antiquités du Maroc, 1961).

[38] Clark, J. D. The earlier Stone Age/Lower Palaeolithic in North Africa and the Sahara. In *New light on the northern African past* (eds. Klees, F. & Kuper, R.) 17–37 (1992).

[39] Mattingly, D. J., Reynolds, T. & Dore, J. N. Synthesis of human activities in the Fazzan. In *The Archaeology of Fazzan* (eds. Mattingly, D. J., Dore, J. N. & Wilson, A. I.) 327–375 (Society for Libyan Studies, 2003).

[40] Reynolds, T. The importance of Saharan lithic assemblages. In *Environment, Climate and Resources of the Libyan Sahara* (eds. Mattingly, D. J., McLaren, S., Savage, E., Al-Fasatwi, Y. & Gadgood, K.) 81–90 (Society for Libyan Studies, 2006).

[41] Boudad L, Guislain S (2012). Acquisition de supports prédéterminés destinés à la réalisation de bifaces: l’exemple de sites de surfaces du Sud-Est marocain. Anthropologie..

[42] Parenti F, Mengoli D, Natali L (2015). The Stone Age in Northwestern Libya: Observations Along a Pipeline. African Archaeol. Rev..

[43] Denys C, Patou M, Djemmali N (1984). Tighennif (Ternifine, Algérie). Premiers résultats concernant l’origine de l’accumulation du matériel osseux de ce gisement Pléistocène. Comptes Rendus l’Academie Sci. - Ser. II.

[44] Geraads D (1986). The Pleistocene Hominid site of Ternifine, Algeria: New results on the environment, age and human industries. Quat. Res..

[45] Raynal J-P, Sbihi-Alaoui F-Z, Geraads D, Magoga L, Mohib A (2001). The earliest occupation of North-Africa: the Moroccan perspective. Quat. Int..

[46] Raynal JP (2010). Hominid Cave at Thomas Quarry I (Casablanca, Morocco): Recent findings and their context. Quat. Int..

[47] Daujeard C, Geraads D, Gallotti R, Mohib A, Raynal J-P (2012). Carcass Acquisition and Consumption by Carnivores and Hominins in Middle Pleistocene Sites of Casablanca (Morocco). J. Taphon..

[48] Daujeard C (2016). Pleistocene hominins as a resource for carnivores: A c. 500,000-year-old human femur bearing tooth-marks in North Africa (Thomas Quarry I, Morocco). PLoS One.

[49] Richter D (2017). The age of the hominin fossils from Jebel Irhoud, Morocco, and the origins of the Middle Stone Age. Nature.

[50] Raynal J-P (1993). La grotte des Rhinocéros (Carrière Oulad Hamida 1, anciennement Thomas III, Casablanca), nouveau site acheuléen du Maroc atlantique. Comptes Rendus l’Académie des Sci. II.

[51] Rhodes E, Raynal J-P, Geraads D, Sbihi-Alaoui F-Z (1994). Premières dates RPE pour l’Acheuléen du Maroc atlantique (Grotte des Rhinocéros, Casablanca). Comptes Rendus l’Academie Sci. - Ser. II.

[52] Rhodes EJ, Singarayer JS, Raynal JP, Westaway KE, Sbihi-Alaoui FZ (2006). New age estimates for the Palaeolithic assemblages and Pleistocene succession of Casablanca, Morocco. Quat. Sci. Rev..

[53] Raynal, J.-P. & Mohib, A. *Préhistoire de Casablanca. I – La Grotte des Rhinocéros (fouilles 1991 et 1996)*. (V.E.S.A.M., 2016).

[54] Lefèvre D, Raynal JP (2009). Les formations plio-pléistocènes de Casablanca et la chronostratigraphie du Quaternaire marin du Maroc revisitées / The Plio-Pleistocene formations of Casablanca and the marine Quaternary chronostratigraphy of Morocco revisited. Quaternaire.

[55] Geraads D (2016). Pleistocene Carnivora (Mammalia) from Tighennif (Ternifine), Algeria. Geobios.

[56] Domínguez-Rodrigo, M., Barba, R. & Egeland, C. P. Deconstructing Olduvai. (Springer, 2007).

[57] Domínguez-Rodrigo M (2002). Hunting and scavenging by the early hominids: the state of the debate. J. World Prehistory.

[58] Domínguez-Rodrigo M, Pickering TR (2003). Early Hominid Hunting and Scavenging: A Zooarcheological Review. Evol. Anthropol..

[59] Bunn HT (1986). Systematic Butchery by Plio/Pleistocene Hominids at Olduvai Gorge, Tanzania [and Comments and Reply]. Curr. Anthropol..

[60] Sahnouni M (2013). The first evidence of cut marks and usewear traces from the Plio-Pleistocene locality of El-Kherba (Ain Hanech), Algeria: Implications for early hominin subsistence activities circa 1.8 Ma. J. Hum. Evol..

[61] Sahnouni M (2018). 1.9-million- and 2.4-million-year-old artifacts and stone tool–cutmarked bones from Ain Boucherit, Algeria. Science (80-.)..

[62] Potts, R. & Shipman, P. Cutmarks made by stone tools on bones from Olduvai Gorge, Tanzania. *Nature* **291** (1981).

[63] Bunn HT (1994). Early Pleistocene hominid foraging strategies along the ancestral Omo River at Koobi Fora, Kenya. J. Hum. Evol..

[64] de Juana S, Galán AB, Domínguez-Rodrigo M (2010). Taphonomic identification of cut marks made with lithic handaxes: An experimental study. J. Archaeol. Sci..

[65] Schlanger, S. Recognizing persistent places in Anasazi Settlement systems. In *Space, Time, and Archaeological Landscapes* (eds. J. Rossignol & L. Wandsnider) 91–112 (Springer, 1992).

[66] Tryon CA (2014). Sites on the landscape: Paleoenvironmental context of late Pleistocene archaeological sites from the Lake Victoria basin, equatorial East Africa. Quat. Int..

[67] Shaw A (2016). The archaeology of persistent places: the Palaeolithic case of La Cotte de St Brelade, Jersey. Antiquity.

[68] Pope, M., McNabb, J. & Gamble, C. Crossing the human threshold. Dynamic transformation and persistent places during the Middle Pleistocene. (Routledge, 2018).

[69] Bernoussi, R. La faune de vertébrés du Pléistocène moyen de la Grotte des Rhinocéros, Casablanca, Maroc: Approche taphonomique préliminaire. In Préhistoire de Casablanca. I – La Grotte des Rhinocéros (fouilles 1991 et 1996) (eds. Raynal, J.-P. & Mohib, A.) 141–143 (V.E.S.A.M., 2016).

[70] Binford, L. R. Faunal Remains from Klasies River Mouth. (Academic Press New York, 1984).

[71] Lam YM, Chen X, Pearson OM (1999). Intertaxonomic Variability in Patterns of Bone Density and the Differential Representation of Bovid, Cervid, and Equid Elements in the Archaeological Record. Am. Antiq..

[72] Lyman, R. L. Vertebrate taphonomy. (Cambridge University Press, 1994).

[73] Munro ND, Bar-Oz G, Stutz AJ (2009). Aging mountain gazelle (Gazella gazella): refining methods of tooth eruption and wear and bone fusion. J. Archaeol. Sci..

[74] Bunn HT, Pickering TR (2010). Methodological recommendations for ungulate mortality analyses in paleoanthropology. Quat. Res..

[75] Hitchins PM (1978). Age determination of the black rhinoceros (Diceros bicornis Linn.) in Zululand. South African. J. Wildl. Res..

[76] Tong H (2001). Age profiles of Rhino Fauna from the middle pleistocene nanjing man site, South China - Explained by the Rhino specimens of living species. Int. J. Osteoarchaeol..

[77] Villa P, Mahieu E (1991). Breakage patterns of human long bones. J. Hum. Evol..

[78] Blumenschine RJ, Selvaggio MM (1988). Percussion marks on bone surface as a new diagnostic of hominid behaviour. Nature.

[79] Pickering TR, Egeland CP (2006). Experimental patterns of hammerstone percussion damage on bones: Implications for inferences of carcass processing by humans. J. Archaeol. Sci..

[80] Vettese, D. *et al*. Towards an understanding of hominin marrow extraction strategies: a proposal for a percussion mark terminology. *Archaeol. Anthropol. Sci.*10.1007/s12520-019-00972-8 (2020).

[81] Blumenschine RJ, Marean CW, Capaldo SD (1996). Blind tests of inter-analyst correspondence and accuracy in the identification of cut marks, percussion marks, and carnivore tooth marks on bone surfaces. J. Archaeol. Sci..

[82] Behrensmeyer AK (1978). Taphonomic and Ecologic Information from Bone Weathering. Palaeobiology.

[83] Domínguez-Rodrigo M, de Juana S, Galán AB, Rodríguez M (2009). A new protocol to differentiate trampling marks from butchery cut marks. J. Archaeol. Sci..

[84] Behrensmeyer AK, Gordon KD, Yanagi GT (1986). Trampling as a cause of bone surface damage and pseudo-cutmarks. Nature.

[85] Brain, C. K. The Hunters or the Hunted? An Introduction to African Cave Taphonomy. (University of Chicago Press, 1981).

[86] Binford, L. R. Bones: ancient men and modern myths. (Academic Press New York, 1981).

[87] Haynes G (1983). A guide for differentiating mammalian carnivore taxa responsible for gnaw damage to herbivore limb bones. Paleobiology.

[88] Olsen SL, Shipman P (1988). Surface modification on bone: Trampling versus butchery. J. Archaeol. Sci..

[89] Blumenschine RJ (1988). An Experimental Model of the Timing of Hominid and Carnivore Influence on Archaeological Bone Assemblages. J. Archaeol. Sci..

[90] Selvaggio MM, Wilder J (2001). Identifying the involvement of multiple carnivore taxa with archaeological bone assemblages. J. Archaeol. Sci..

[91] Domínguez-Rodrigo M, Piqueras A (2003). The use of tooth pits to identify carnivore taxa in tooth-marked archaeofaunas and their relevance to reconstruct hominid carcass processing behaviours. J. Archaeol. Sci..

[92] Pickering TR, Domínguez-Rodrigo M, Egeland CP, Brain CK (2004). Beyond leopards: Tooth marks and the contribution of multiple carnivore taxa to the accumulation of the Swartkrans Member 3 fossil assemblage. J. Hum. Evol..

[93] Tong HW, Zhang S, Chen F, Li Q (2008). Rongements sélectifs des os par les porcs-épics et autres rongeurs: cas de la grotte Tianyuan, un site avec des restes humains fossiles récemment découvert près de Zhoukoudian (Choukoutien). Anthropologie.

[94] Blasco R, Rosell J, Fernández Peris J, Cáceres I, Vergès JM (2008). A new element of trampling: an experimental application on the Level XII faunal record of Bolomor Cave (Valencia, Spain). J. Archaeol. Sci..

[95] Shipman P, Rose JJ (1984). Cutmark Mimics on Modern and Fossil Bovid Bones. Curr. Anthropol..

[96] De Heinzelin J (1999). Environment and behavior of 2.5-million-year-old Bouri hominids. Science (80-.)..

[97] Fiore I (2004). Taphonomic analysis of the Late Early Pleistocene bone remains from Buia (Dandiero Basin, Danakil depression, Eritrea): Evidence for large mammal and reptile butchering. Riv. Ital. di Paleontol. e Stratigr..

[98] Domínguez-Rodrigo M, Pickering TR, Semaw S, Rogers MJ (2005). Cutmarked bones from Pliocene archaeological sites at Gona, Afar, Ethiopia: Implications for the function of the world’s oldest stone tools. J. Hum. Evol..

[99] Lyman, R. L. Quantitative paleozoology. (Cambridge University Press, 2008).

[100] Brain, C. K. Some principles in the interpretation of bone accumulations associated with man. in Human origins, Louis Leakey and the East African Evidence (eds. Isaac, G. & McCown, E.) 96–116 (W. A. Benjamin Advanced Book Program, 1976).

[101] White, T. D. Prehistoric Cannibalism at Mancos 5MTUMR-2346. (Princeton University Press, 1992).

[102] Fernández-Jalvo Y, Díez JC, Cáceres I, Rosell J (1999). Human cannibalism in the Early Pleistocene of Europe (Gran Dolina, Sierra de Atapuerca, Burgos, Spain). J. Hum. Evol..

[103] Fernández-Jalvo Y, Andrews P (2011). When humans chew bones. J. Hum. Evol..

[104] Bello SM, Saladié P, Cáceres I, Rodríguez-Hidalgo A, Parfitt SA (2015). Upper Palaeolithic ritualistic cannibalism at Gough’s Cave (Somerset,UK): THE human remains from head to toe. J. Hum. Evol..

[105] Saladié P, Rodríguez-Hidalgo A, Díez C, Martín-Rodríguez P, Carbonell E (2013). Range of bone modifications by human chewing. J. Archaeol. Sci..

[106] Grün R, Schwarcz HP, Chadam JM (1988). ESR dating of tooth enamel: coupled correction for U-uptake and U-series disequilibrium. Nucl. Tracks Radiat. Meas..

[107] Shao Q, Bahain J-J, Falguères C, Dolo JM, Garcia T (2012). A new U-uptake model for combined ESR/U-series dating of tooth enamel. Quat. Geochronol..

[108] Grün R (2006). Direct dating of human remains. Yearb. Phys. Anthropol..

[109] Falguères C (2010). A 300-600 ka ESR/U-series chronology of Acheulian sites in Western Europe. Quat. Int..

[110] Bischoff JL, Rosenbauer RJ, Tavoso A, de Lumley H (1988). A test of uranium-series dating of fossil tooth enamel: Results from Tournal cave. France. Appl. Geochemistry.

[111] Pons-Branchu E, Hillaire-Marcel C, Deschamp P, Ghaleb B, Sinclair DJ (2005). Early diagenesis impact on precise U-series dating of deep-sea corals: example of a 100-200-years old Lophelia pertusa sample from the northeast Atlantic. Geochim. Cosmochim. Acta.

[112] Yokoyama, Y. & Nguyen, H. V. Direct and non destructive dating of marine sediments, manganese nodules and corals by high resolution gamma-ray spectrometry. in Isotope marine chemistry (ed. Goldberg, E. D.) 259–289 (Geochemistry Research Association, 1980).

[113] Grün R, Joannes-Boyau R, Stringer C (2008). Two types of CO2− radicals threaten the fundamentals of ESR dating of tooth enamel. Quat. Geochronol..

[114] Grün R (2009). The DATA program for the calculation of ESR age estimates on tooth enamel. Quat. Geochronol..

[115] Grün R, Katzenberger-Apel O (1994). An alpha irradiator for ESR dating. Anc. TL.

[116] Brennan BJ, Rink WJ, McGuirl EL, Schwarcz HP, Prestwich WV (1997). Beta doses in tooth enamel by ‘“One Group”’ theory and the Rosy ESR dating software. Radiat. Meas..

[117] Guérin G, Mercier N, Nathan R, Adamiec G, Lefrais Y (2012). On the use of the infinite matrix assumption and associated concepts: A critical review. Radiat. Meas..

[118] Bahain J-J, Yokoyama Y, Falguères C, Sarcia MN (1992). ESR dating of tooth enamel: a comparison with K-Ar dating. Quat. Sci. Rev..

[119] Hublin JJ (2017). New fossils from Jebel Irhoud, Morocco and the pan-African origin of Homo sapiens. Nature.

