# Supplementary material for: Earliest African evidence of carcass processing and consumption in cave at 700 ka, Casablanca, Morocco
Source: Sci Rep. 2020 Mar 16;10:4761. doi: 10.1038/s41598-020-61580-4 (PMC7075909; doi:10.1038/s41598-020-61580-4)
Supplement: Supplementary file 1 — Supplementary Information. [file 41598_2020_61580_MOESM1_ESM.pdf]

SUPPLEMENTARY INFORMATION

Earliest African evidence of carcass processing and consumption in cave at 700 ka, Casablanca, Morocco

Camille Daujeard[1]*, Christophe Falguères[1], Qingfeng Shao[2], Denis Geraads[3,4], Jean-Jacques Hublin[4,5], David Lefèvre[6], Mohssine El Graoui[7], Mathieu Rué[6,8], Rosalia Gallotti[6,9], Vincent Delvigne[9,10], Alain Queffelec[9], Eslem Ben Arous[1], Olivier Tombret[1], Abderrahim Mohib[7,11], Jean-Paul Raynal[9,4]

[1] HNHP-UMR 7194, CNRS, MNHN, UPVD, Sorbonne Universités, Institut de Paléontologie Humaine, 1 rue René Panhard, 75013 Paris, France. camille.daujeard@mnhn.fr

*** Corresponding author**

[2] College of Geography Science, Nanjing Normal University, 1, Wenyuan Road, 210023 Nanjing, China

[3] CR2P-UMR 7207, CNRS, MNHN, Sorbonne Universités, CP 38, 8 rue Buffon, 75231 Paris cedex 05, France

[4] Department of Human Evolution, Max Planck Institute for Evolutionary Anthropology, Deutscher Platz 6, 04103 Leipzig, Germany

[5] Collège de France, 11 place Marcelin Berthelot, 75005 Paris, France

[6] Université Paul Valéry Montpellier 3, CNRS, UMR 5140 Archéologie des sociétés méditerranéennes, Campus Saint Charles, 34199 Montpellier, France

[7] Institut National des Sciences de l'Archéologie et du Patrimoine (INSAP), Madinat al-Irfane, les Instituts - Hay Riyad, B.P. 6828, 10100 Rabat, Morocco

[8] Paléotime SARL, 6173 avenue JS Achard-Picard, 38250 Villard-de-Lans, France

[9] Université de Bordeaux, CNRS, UMR 5199 PACEA, Bâtiment B2, allée Geoffroy Saint-Hilaire, CS 50023, 33615 Pessac cedex, France

[10] Service de Préhistoire, Université de Liège, place du XX août, 4000 Liège, Belgium

[11] Direction provinciale de la Culture, Avenue Mohammed V, quartier administratif, Kénitra, Morocco.

This PDF file includes:
- Supplementary text
- Supplementary references
- Figures S1 to S5
- Tables S1 to S10

SUPPLEMENTARY INFORMATION (TEXT)

1. The Oulad Hamida Formation

The Casablanca hinterland record an exceptional succession of palaeoshorelines since the end of the Miocene[1–4]. From the late Early to the Upper Pleistocene, four Formations associated with four raised platforms and including several Members have been defined: the Oulad Hamida, the Anfa, the Kef El Haroun, and the Dar Bou Azza Formations[4–5].

The Oulad Hamida Formation (OHF) is based on a major unconformity formed at an altitude of around 28 m above sea level (asl) at the expense of the Cretaceous or Palaeozoic substratum, 10 m above the platform of the Anfa Formation. OHF include five members (OH1 to OH5)[5,6,7]. According to the sequence stratigraphy, each member is an allostratigraphy unit defined by a unitary sequence, characterized by an association and succession of genetically related deposits bounded at its base and top by unconformities. Each sequence/member records transgressive and highstand tracks systems corresponding to high sea levels associated with the formation of a shoreline marked by a cliff in the case of OH3 and OH4. The unconformities or sequence boundaries that separate them sign the regression of the ocean. These Members are thus correlative of cyclic sea level fluctuations in relation to global glacio-eustatic changes, preserved thanks to regional tectonic uplift. The OHF predates the Anfa Formation whose Members correlate to MIS 11, 13 and 15[3,7–9]. Thus, the OHF records at least four sea-level highstand, from the late Early Pleistocene to the beginning of the Middle Pleistocene, before MIS 15.

GDR belongs to a palaeoshoreline with cliff and deep cavities which constitute the more recent member (OH4) of the OHF. The continental deposits complex in GDR post-date OH4.

2. Sedimentary infilling of GDR and site formation processes

The 7 m thick infilling sequence consists of a superposition of yellowish and reddish calcarenitic units observed in the bottom of the Grotte des Rhinocéros (GDR) (Figure S1a). This sequence has been divided into two lithostratigraphic sets separated by a gradual transition. The lower set (units 5 and 6) is characterized by 1) the presence of calcarenitic megablocks from the collapse of the ceiling and 2) the prevalence of the reddish units. The upper set (units 1 to 4) shows a more regular stratification dominated

by the yellowish units. In both sets, the stratification is flat with a regular dip towards the north and the bottom of the cave (3° at most). The sedimentary filling was studied by using a geoarchaeological approach crossed on the sediment material (micromorphology, particle size analysis, ED-XRF and XRD analysis) and the lithic and faunal plotted material (spatial analysis, particle size distribution, fabric analysis). In the reddish deposits, were faunal remains and lithic objects are more numerous, microfacies observed in thin sections show sandy dune material mixed with particles coming from the erosion of red soils around the cavity, locally run off at the bottom of the cave (Figure S1b). In the yellowish units, microfacies suggest that sediment mainly corresponds to sandy dune material blown into the cavity (Figure S1c). Thin sections show runoff and decantation structures suggesting low-energy water transport (Figure S1b). This alternation of facies records local or general variations in sedimentation (possibly under climatic control). Large elements (cobbles) are rare and may result from anthropogenic (manuports) or natural inputs by reworking of previous coarse marine deposits. After the infilling, three main diagenetic phases have affected this sequence (alteration, cementation then dissolution)[10]. Flat stratification excludes important post-depositional phenomena (such as suffosion).

Due to the diagenetic evolution, the initial texture of the sediment is difficult to appreciate. The granulometric analysis reveals a plurimodal silty sand (protocol according to Sitzia et al.[11]). The main mode (around 300 μm) derives from the calcarenitic bedrock (Figure S1d). The lower modes have more complex origin (aeolian contributions, eroded soils, etc.). In any cases, the lithics and the faunal remains are currently in contact with a loamy coarse sand (maximum diameter around 1 mm). XRD analysis was made on bulk powdered samples with a Malvern Panalytical XPERT-PRO diffractometer using a Cu K-alpha radiation at 30 mA, 45 kV. Samples were treated in the range from 10 to 80° 2Th with a step size of 0.033° 2Th and a scan step time of 69.85 s. Results, based on Rietveld refinement using HighScore Plus software, give contents of calcite and quartz respectively of approximately 70 and 30% for unit 5 (sample 23, Extended Data Fig. 2a).

The fabrics analysis (protocol according to Lenoble et al.[12]) confirms that the faunal remains and lithics in the lower set are moderately affected by runoff (Figure S1e). The spatial distribution of plotted objects is not homogenous. Concentrations have been identified in the reddish facies, especially in the lower set where fauna elements are relatively more numerous. In this lower part, megablocks and Rhinoceros skulls, as well as other large mammal elements, have probably constrained the distribution of the

material. The surface states of the bone and lithic material show moderate mechanical alteration stigmata indicative of *in situ* reworking, in agreement with sedimentary data (low energy inputs). The process of redistribution is probably mainly due to the activities of the fauna in the cavity.

3. GDR and the Second Regional Acheulean at Casablanca

The assemblage of GDR was ascribed to the Second Regional Acheulean (SRA)[13,14]. It has been manufactured mainly with different varieties of quartzite and a few flint nodules[15]. The macro-industry comprises handaxes of various morphologies and dimensions, as well as rare cleavers and pebble tools[16]; the micro-industry is mainly made of raw flakes that, apart from those coming from the shaping of bifacial pieces, were produced by discoid (unifacial and bifacial) and polyhedric (multifacial) flaking, not any evidence of Levallois flaking has for now been identified; retouched flakes are rare, notches and denticulates are a majority and little diversified[17] (Figures S2). This is a rather banal and frequent composition described in the literature for various Acheulean sites in Africa but also Middle Pleistocene European ones.

Most of the SRA sites at Casablanca were found in caves and a few open-air sites within Middle Pleistocene units: Sidi Abderrahmane Quarry (Grotte des Ours, Grotte des Littorines and Cap Chatelier), Sidi Abderrahmane Extension Quarry, Sidi Al Khaddir (former Helaoui Quarry), Thomas Quarry I (Grotte à Hominidés, GH) and Oulad Hamida 1 Quarry (GDR). Assemblages from caves (GH and GDR upper set) are rich in small flaking, cores and core tools (including flaked cobbles with SSDA method) and associated with an abundant fauna in which medium and small size mammals have been hunted and scavenged. Some knapping workshops exist in open-air localities (Sidi Al Khadir-Helaoui), but may be so-called considering the absence of preserved faunal remains. The SRA is very polymorph, represented as soon as 0.5-0.7 Ma in GH at Thomas I Quarry and in GDR. It develops onwards to MIS 11 represented at Sidi Abderrahmane by the Grotte des Ours and Grotte des Littorines assemblages. Its more recent terms show a common use of Levallois flaking, prior to 367 ± 34 ka at Cap Chatelier (D2) and younger than 330 ± 34 ka at Sidi Abderrahmane-Extension upper layer and is apparently contemporaneous with the earliest known Middle Stone Age of the hinterland[18].

4. Geochronology

Table 1 shows the uranium content and isotopic ratios of the different tissues for each tooth (Figure S3a). The samples have been analysed by alpha spectrometry in the Paris lab (UMR 7194, CNRS, France). For the lower area, U content is comprised between 0.78 and 1.11 ppm and between 14.76 and 20 ppm while in the upper area, U content is ranging between 3.3 and 5.6 ppm. U content in dentine varies between 58 and 85 ppm. $^{234}U/^{238}U$ ratio ranges between 1.170 and 1.447 ppm for enamel and between 1.381 and 1.571 ppm for dentine. The $^{230}Th/^{234}U$ for enamel is comprised between 0.918 and 1.114 ppm. Only one ratio (GDR5244) shows real leaching process. For dentine, it is exactly the opposite, only the GDR5244 does not show any uranium loss and all the others are beyond the unity.

Table S2 shows Equivalent doses (DE), annual dose-rate divided into internal and sediment + cosmic contributions, n and p-values and ages of the teeth (Figure S3b). DE range between 784 and 3382 Gy for the lower set while DE is comprised between 1046 and 1615 Gy for the upper set. As mentioned previously, DE were fitted with an EXPLIN function because a single SSE overestimates the results from 5 to 30% according the samples. Though[19] recommend a DSE function to fit samples with a high DE range (more than 2000 Gy), it seems that in the case of our samples, an EXPLIN function being more appropriate and presenting slight better r2 adjustment and smaller error range excepting for GDR8071. Figure S3c shows a linear correlation between enamel U content and the age of samples coming from the lower set. The intersection with abscissa suggests the moment of the burial of the teeth, when the tissues have no uranium, around 715 ka. This result is in agreement with the 690-720 ka age range obtained for the GDR6818 and GDR7067 samples.

Because cement tissue was absent in all the teeth, a Dentine-Enamel-Sediment system was adopted for analyse. Figures S3d shows the proportions between internal (alpha + beta contribution in enamel) and external (beta + gamma contribution in the sediment plus cosmic) doses for each tooth. For the lower set, internal dose is more than 60% of the total dose and reaching more than 90% for bovid teeth. For the upper set, a high percentage for internal dose is also reached (more than 80% of the total dose). The uptake uranium values show that enamel underwent a general post-mortem uptake except for GDR7433 in the lower part of the site and in the upper part where teeth got a U-leaching. On the other hand, dentine underwent systematically uranium leaching apart GDR5244 which underwent a recent uranium uptake. This traduces the complexity of the geochemical history of this site.

5. Biochronology

The vertebrates from GDR were recently studied in detail[20–29]; only their major features are listed below. Because North-western small mammals are strongly endemic, most comparisons cannot extend beyond this region. The main reference sites are the nearby loci of Thomas I Quarry[30–32], and those studied long ago by[33–35], especially Tighennif in Algeria, probably dating to the late lower Pleistocene, c. 1 Ma[36]. The assemblage from GDR is generally similar to that of the main assemblage from Thomas I (called ThI-ABCE by[30–32]) but there are some indications that it is slightly younger than the one from the hominid site ThI-GH. However, these indications are partly based upon size differences, usually assumed to be time-related, but which might also stem from climatic or other environmental changes.

Perhaps the most unexpected taxon of GDR is a lagomorph, *Trischizolagus ?raynali*, first assigned to the genus *Serengetilagus*[37], defined upon *S. praecapensis* from Laetoli. Its genus assignment was questioned by[38], who referred it to the Mediterranean genus *Trischizolagus* instead. The affinities of the species remain debatable, because its characteristics might just result from its age, much younger than all other species of these genera but, in any case, it puts an ancient stamp on the fauna.

The best specimen of the cercopithecid primate *Theropithecus oswaldi* is a partial mandible from nearby, probably contemporaneous fissure fillings, but the species is quite rare at GDR itself. A well-preserved M3 is larger than those from the Okote Member of Koobi Fora, and even than the rare specimens from Tighennif. Even if the correlation between size and age is not straightforward (as shown by the large size of teeth from Olduvai Bed II), this certainly suggests a relatively recent age in the Pleistocene. This is confirmed by the small size of the lower canines, which underwent reduction in this lineage, but the species is not definitely known after c. 0.5 Ma in East Africa.

The numerous carnivores yield little biochronological information. Both species of hyenas, and the wildcat, are still extant, but they were already present at Tighennif. Two other felids, instead, are certainly distinct from living ones: these are *Lynx thomasi*, found in nearby fissure fillings, but unknown elsewhere, and a *Panthera* intermediate in size between the leopard and the lion, reminiscent of the European *P. gombaszogensis*, but whose identity cannot be definitely established. Sabertooth cats, still present (albeit quite rare) at Tighennif, are missing at GDR. Among canids, the abundant North-African endemic extinct omnivorous *Lupulella mohibi* has a broad chronological range, from Tighennif to Sidi Abderrahmane[39], while the rare hunting-dog *Lycaon* is probably distinct from the living form. The bear

is definitely also of an extinct species, but the evolution of *Ursus* in North-Africa remains imperfectly known.

The remarkably abundant *Ceratotherium* belongs to a species close to, but distinct from, the modern 'white' rhino; its extinction in North-Africa certainly post-dates its extinction in Eastern Africa. Yet, its presence is an archaic feature of the fauna.

The zebra *Equus* cf. *mauritanicus* is probably close to, but distinct from the Tighennif form, but the origin of the difference might not be chronological.

Suids clearly differ from those of Tighennif, with the extant warthog *Phacochoerus africanus* replacing the larger (but probably ecologically similar) *Metridiochoerus compactus*.

Bovids are also clearly distinct but, again, the geographic location and environments of the sites probably account for a large part of the differences. Although poorly known, the most common bovid at GDR is reminiscent of an alcelaphin present in the Okote Member of Koobi Fora.

Faunal list

Squamata

Timon pater (LATASTE, 1880)

Malpolon monspessulanus (HERMANN, 1804)

Hemorrhois hippocrepis (L.)

Aves

Phoebastria albatrus (PALLAS, 1769)

Struthio camelus L.

Morus bassanus (L.)

Anas sp. (*A. strepera* L. or *A.penelope* L.)

Aythya cf. *marila* L.

Alectoris cf. *barbara* (BONNATERRE, 1791)

Coturnix coturnix (L.)

Pterocles orientalis (L.)

Columba livia GMELIN, 1789

Strix aluco L.

Apus sp.

Corvus monedula L.

Small passeriforms

Rodentia

Mus hamidae GERAADS, 1994

Praomys darelbeidae GERAADS, 1994

Paraethomys tighenifae JAEGER, 1975

Gerbillus grandis minor GERAADS, 1994

Dipodillus cf. *campestris* (LOCHE, 1867)

Meriones shawi hamidae GERAADS, 1994

Meriones maximus TONG, 1986

Ellobius atlanticus JAEGER, 1975

Eliomys darelbeidae GERAADS, 1994

Hystrix aff. *cristata* L.

Lagomorpha

Trischizolagus ? *raynali* (GERAADS, 1994)

Lepus cf. *capensis* L.

Insectivora

Crocidura darelbeidae GERAADS, 1993

Crocidura cf. *tarfayaensis* VESMANIS & VESMANIS, 1979

Cercopithecidae

Theropithecus oswaldi ANDREWS, 1916

Carnivora

Hyaena hyaena L.

Crocuta crocuta ERXLEBEN, 1777

Panthera cf. *gombaszogensis* KRETZOI, 1938

Felis silvestris SCHREBER, 1777

Lupulella mohibi GERAADS, 2011

Vulpes cf. *rueppelli* SCHINZ, 1825

Lycaon cf. *magnus* EWER & SINGER, 1956

Mellivora capensis SCHREBER, 1776

Ursus cf. *bibersoni* ENNOUCHI, 1957

Perissodactyla

Ceratotherium mauritanicum (POMEL, 1888)

Equus cf. *mauritanicus* POMEL, 1888

Artiodactyla

Phacochoerus cf. *africanus* GMELIN, 1788

Camelus cf. *thomasi* POMEL, 1893

Cf. *Syncerus antiquus* (DUVERNOY, 1851)

Oryx sp.

cf. *Parmularius* sp.

Connochaetes taurinus prognu POMEL, 1894

Gazella cf. *atlantica* BOURGUIGNAT, 1870

Supplementary References

1. Biberson, P. *Le Paléolithique inférieur du Maroc atlantique*. (Publications du Service des Antiquités du Maroc, 1961).
2. Stearns, C. E. Pliocene-Pleistocene emergence of the Moroccan Meseta. *Geol. Soc. Am. Bull.* **89**, 1630–1644 (1978).
3. Lefèvre, D. & Raynal, J.-P. Les formations Plio-Pléistocènes de Casablanca et la chronostratigraphie du Quaternaire marin du Maroc revisité. *Quaternaire* **13**, 9–21 (2002).
4. Texier, J., Lefèvre, D. & Raynal, J.-P. Contribution pour un nouveau cadre stratigraphique des formations littorales quaternaires de la région de Casablanca. *Comptes Rendus l'Academie Sci. - Ser. II* **318**, 1247–1253 (1994).
5. Lefèvre, D., Raynal, J.-P. & El Graoui, M. Le Quaternaire d'Oulad Hamida. in *Préhistoire de Casablanca. I – La Grotte des Rhinocéros (fouilles 1991 et 1996)* (eds. Raynal, J.-P. & Mohib, A.) 45–59 (V.E.S.A.M., 2016).
6. Lefèvre, D. & El Graoui, M. Les formations pléistocènes de la carrière Oulad Hamida 1. in *Préhistoire de Casablanca. I – La Grotte des Rhinocéros (fouilles 1991 et 1996)* (eds. Raynal, J.-P. & Mohib, A.) 61–77 (V.E.S.A.M., 2016).
7. Lefèvre, D. Du continent à l'océan. Morphostratigraphie et paléogéographie du Quaternaire du Maroc atlantique. Le modèle casablancais. (Montpellier 3, 2000).
8. Rhodes, E. J., Singarayer, J. S., Raynal, J. P., Westaway, K. E. & Sbihi-Alaoui, F. Z. New age estimates for the Palaeolithic assemblages and Pleistocene succession of Casablanca, Morocco. *Quat. Sci. Rev.* **25**, 2569–2585 (2006).
9. Texier, J.-P., Lefèvre, D., Raynal, J.-P. & Graoui, M. El. Lithostratigraphy of the littoral deposits of the last one million years in Casablanca region (Maroc). *Quaternaire* **13**, 23–41 (2002).
10. Raynal, J.-P., Lefèvre, D., El Graoui, M., Geraads, D. & Rué, M. La Grotte des Rhinocéros (Casablanca, Maroc) : le remplissage et son âge. in *Préhistoire de Casablanca. I – La Grotte des Rhinocéros (fouilles 1991 et 1996)* (eds. Raynal, J.-P. & Mohib, A.) 79–86 (V.E.S.A.M., 2016).
11. Sitzia, L. *et al.* Dynamics and sources of last glacial aeolian deposition in southwest France derived from dune patterns, grain-size gradients and geochemistry, and reconstruction of efficient wind directions. *Quat. Sci. Rev.* **170**, 250–268 (2017).
12. Lenoble, A. & Bertran, P. Fabric of Palaeolithic levels: Methods and implications for site formation processes. *J. Archaeol. Sci.* **31**, 457–469 (2004).
13. Raynal, J.-P. & Mohib, A. *Préhistoire de Casablanca. I – La Grotte des Rhinocéros (fouilles 1991 et 1996)*. (V.E.S.A.M., 2016).
14. Raynal, J.-P., Gallotti, R., Mohib, A., Fernandes, P. & Lefèvre, D. The western quest, First and Second Regional Acheuleans at Thomas-Oulad Hamida Quarries (Casablanca, Morocco). in *Vocation préhistoire. Hommage à Jean-Marie Le Tensorer* . (eds. Woitczak, D. et al.) 309–322 (ERAUL, 2017).
15. Fernandes, P., Raynal, J.-P. & Mohib, A. L'exploitation des ressources minérales par les hommes du Second Acheuléen régional (Grotte des Rhinocéros , Casablanca, Maroc). in *Préhistoire de Casablanca. I – La Grotte des Rhinocéros (fouilles 1991 et 1996)* (eds. Raynal, J.-P. & Mohib, A.) 145–154 (V.E.S.A.M., 2016).
16. Raynal, J.-P., Mohib, A., Magoga, L. & Sbihi-Alaoui, F. Z. La production lithique dans le second Acheuléen régional de la Grotte des rhinocéros (Casablanca, Maroc). 2 : Le macro-outillage. in *Préhistoire de Casablanca. I – La Grotte des Rhinocéros (fouilles 1991 et 1996)* (eds. Raynal, J.-P. & Mohib, A.) 195–210 (V.E.S.A.M., 2016).
17. Raynal, J.-P., Mohib, A., Magoga, L., Krarssi, W. & Sbihi-Alaoui, F. Z. La production lithique dans le second Acheuléen régional de la Grotte des rhinocéros (Casablanca, Maroc) 1 : les éclats et micro-outillage sur éclats. in *Préhistoire de Casablanca. I – La Grotte des Rhinocéros (fouilles 1991 et 1996)* (eds. Raynal, J.-P. & Mohib, A.) 183–193 (V.E.S.A.M., 2016).
18. Richter, D. *et al.* The age of the hominin fossils from Jebel Irhoud, Morocco, and the origins of the Middle Stone Age. *Nature* **546**, 293–296 (2017).
19. Duval, M. & Grün, R. Are published ESR dose assessments on fossil tooth enamel reliable? *Quat. Geochronol.* **31**, 19–27 (2016).
20. Bailón, S. La faune de vertébrés du Pléistocène moyen de la Grotte des Rhinocéros, Casablanca, Maroc : 1 - Les Squamates (Reptilia). in *Préhistoire de Casablanca. I – La Grotte des Rhinocéros (fouilles 1991 et 1996)* (eds. Raynal Jean-Paul & Mohib, A.) 87 (V.E.S.A.M., 2016).
21. Mourer-Chauviré, C. La faune de vertébrés du Pléistocène moyen de la Grotte des Rhinocéros,

Casablanca, Maroc : 2 - Les Oiseaux. in *Préhistoire de Casablanca. I – La Grotte des Rhinocéros (fouilles 1991 et 1996)* (eds. Raynal, J.-P. & Mohib, A.) 89–90 (V.E.S.A.M., 2016).
22. Geraads, D. & Alemseged, Z. La faune de vertébrés du Pléistocène moyen de la Grotte des Rhinocéros, Casablanca, Maroc : 3 - Cercopithecidae. in *Préhistoire de Casablanca. I – La Grotte des Rhinocéros (fouilles 1991 et 1996)* (eds. Raynal, J.-P. & Mohib, A.) 91–93 (V.E.S.A.M., 2016).
23. Geraads, D. La faune de vertébrés du Pléistocène moyen de la Grotte des Rhinocéros, Casablanca, Maroc : 4 - Rongeurs et Lagomorphes. in *Préhistoire de Casablanca. I – La Grotte des Rhinocéros (fouilles 1991 et 1996)* (eds. Raynal, J.-P. & Mohib, A.) 95–104 (V.E.S.A.M., 2016).
24. Geraads, D. La faune de vertébrés du Pléistocène moyen de la Grotte des Rhinocéros, Casablanca, Maroc : 5 - Insectivores. in *Préhistoire de Casablanca. I – La Grotte des Rhinocéros (fouilles 1991 et 1996)* (eds. Raynal, J.-P. & Mohib, A.) 105–110 (V.E.S.A.M., 2016).
25. Geraads, D. & Bernoussi, R. La faune de vertébrés du Pléistocène moyen de la Grotte des Rhinocéros, Casablanca, Maroc : 6 - Carnivora. in *Préhistoire de Casablanca. I – La Grotte des Rhinocéros (fouilles 1991 et 1996)* (eds. Raynal, J.-P. & Mohib, A.) 111–119 (V.E.S.A.M., 2016).
26. Geraads, D. & Bernoussi, R. La faune de vertébrés du Pléistocène moyen de la Grotte des Rhinocéros, Casablanca, Maroc : 7 - Rhinocerotidae. in *Préhistoire de Casablanca. I – La Grotte des Rhinocéros (fouilles 1991 et 1996)* (eds. Raynal, J.-P. & Mohib, A.) 121–127 (V.E.S.A.M., 2016).
27. Geraads, D. & Bernoussi, R. La faune de vertébrés du Pléistocène moyen de la Grotte des Rhinocéros, Casablanca, Maroc : 8 - Equidae. in *Préhistoire de Casablanca. I – La Grotte des Rhinocéros (fouilles 1991 et 1996)* (eds. Raynal, J.-P. & Mohib, A.) 129–131 (V.E.S.A.M., 2016).
28. Geraads, D. & Bernoussi, R. La faune de vertébrés du Pléistocène moyen de la Grotte des Rhinocéros, Casablanca, Maroc : 9 - Hippopotamidae, Suidae et Camelidae. in *Préhistoire de Casablanca. I – La Grotte des Rhinocéros (fouilles 1991 et 1996)* (eds. Raynal, J.-P. & Mohib, A.) 133–134 (V.E.S.A.M., 2016).
29. Geraads, D. & Amani, F. La faune de vertébrés du Pléistocène moyen de la Grotte des Rhinocéros, Casablanca, Maroc : 10 - Bovidae. in *Préhistoire de Casablanca. I – La Grotte des Rhinocéros (fouilles 1991 et 1996)* (eds. Raynal, J.-P. & Mohib, A.) 135–139 (V.E.S.A.M., 2016).
30. Geraads, D. Plio-Pleistocene Mammalian biostratigraphy of Atlantic Morocco. *Quaternaire* **13**, 43–53 (2002).
31. Geraads, D. Biochronologie mammalienne du Quaternaire du Maroc atlantique dans son cadre régional. *Anthropologie.* **114**, 324–340 (2010).
32. Geraads, D., Raynal, J. P. & Sbihi-Alaoui, F. Z. Mammalian faunas from the Pliocene and Pleistocene of Casablanca (Morocco). *Hist. Biol.* **22**, 275–285 (2010).
33. Jaeger, J. J. Les Muridae (Mammalia, Rodentia) du Pliocène et du Pléistocène du Maroc. (Montpellier, 1975).
34. Jaeger, J. J. Origine et évolution du genre Ellobius (Mammalia Rodentia) en Afrique Nord-Occidentale. *Folia Quat.* **57**, 3–50 (1988).
35. Tong, H. The Gerbillinae (Rodentia) from Tighennif (Pleistocene of Algeria) and their significance. *Mod. Geol.* **10**, 197–214 (1986).
36. Geraads, D. Pleistocene Carnivora (Mammalia) from Tighennif (Ternifine), Algeria. *Geobios* **49**, 445–458 (2016).
37. Geraads, D. Rongeurs et Lagomorphes du Pléistocène moyen de la 'Grotte des Rhinocéros', carrière Oulad Hamida 1, à Casablanca, Maroc. *Neues Jahrb. für Geol. und Paläontologie Abhandlungen* **191**, 147–172 (1994).
38. Averianov, A. O. & Tesakov, A. S. Evolutionary trends in Mio-Pliocene Leporidae, based on Trischizolagus (Mammalia, Lagomorpha). *Paläontologische Zeitschrift* **71**, 145–153 (1997).
39. Geraads, D. A revision of the fossil Canidae (Mammalia) of north-western Africa. *Palaeontology* **54**, 429–446 (2011).
40. Brain, C. K. *The Hunters or the Hunted ? An Introduction to African Cave Taphonomy.* (University of Chicago Press, 1981).
41. Brugal, J.-P., Fosse, P. & Guadelli, J. Comparative study of bone assemblages made by recent and pleistocene hyenids. in *Proceedings of the 1993 bone modification conference* (eds. Hannus, L. A., Rossum, L. & Whinam, R. P.) 158–187 (Hot Springs, 1997).

SUPPLEMENTARY INFORMATION (FIGURES)

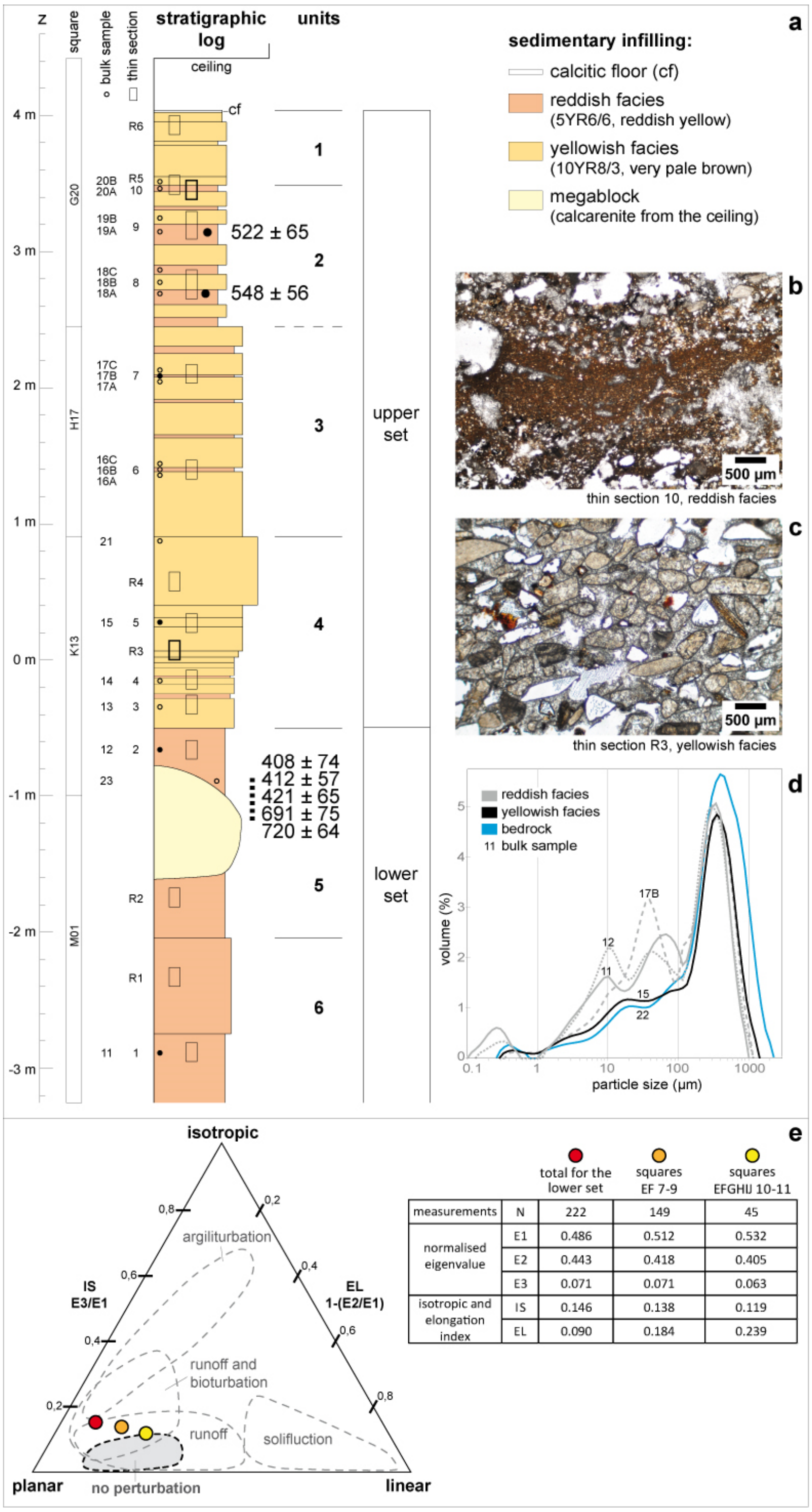

		total for the lower set	squares EF 7-9	squares EFGHIJ 10-11
measurements	N	222	149	45
normalised eigenvalue	E1	0.486	0.512	0.532
	E2	0.443	0.418	0.405
	E3	0.071	0.071	0.063
isotropic and elongation index	IS	0.146	0.138	0.119
	EL	0.090	0.184	0.239

Figure S1. Sedimentary context and results of fabrics analysis. Graphs elaboration and drawings by M. Rué. **a**, Stratigraphic log of the infilling and location of the US-ESR dates (ka). **b-c**, Examples of microfacies of the reddish (b) and yellowish (c) units (Plane-polarized light microscope photos by M. Rué from thin-sections). **d**, Grain-size distribution of some representative samples of the infilling and the bedrock. **e**, Results of fabrics analysis from lower set. Benn's diagram according to[12].

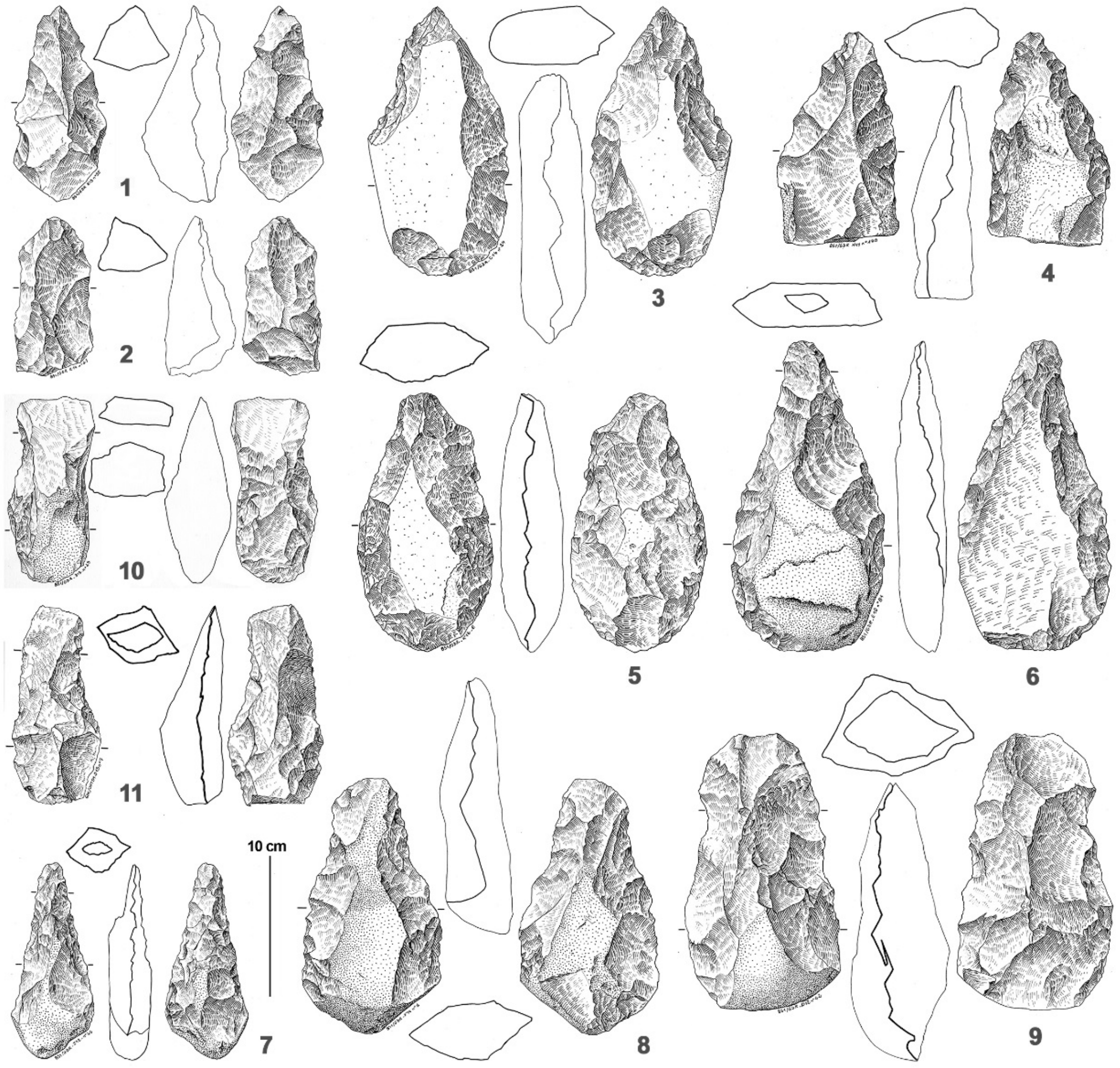

Figure S2. GDR lithic assemblage. Original drawings by M. Hirbec-Raynal.

The lithics found at GDR in the lower set consist of 2041 flakes which were obtained from quartzites (n = 2012) and flint (n = 29) by various flaking methods (289 cores) and are little retouched (132 tools on flakes). The 161 Large Cutting Tools and Heavy Duty Tools are exclusively manufactured on quartzite cobbles and large flakes and this process partly took part outside of the excavated area based on the number of shaping flakes which were retrieved. These large tools include varied choppers, polyhedrons, spheroids, trihedrons and picks (1 and 2), handaxes of various morphologies and dimensions (3 to 9), as well as very rare cleavers (10 and 11). Knappers were particularly skilled to work the quartzites and the morphologies of the LCTs active parts clearly express pre-determined processes aimed at specialized activities. The broken points of handaxes testify to percussion, torsion and bending gestures while the retouched sides point to cutting actions. Moreover, proximal parts of bifaces often bear percussion marks demonstrating their multifunctional role.

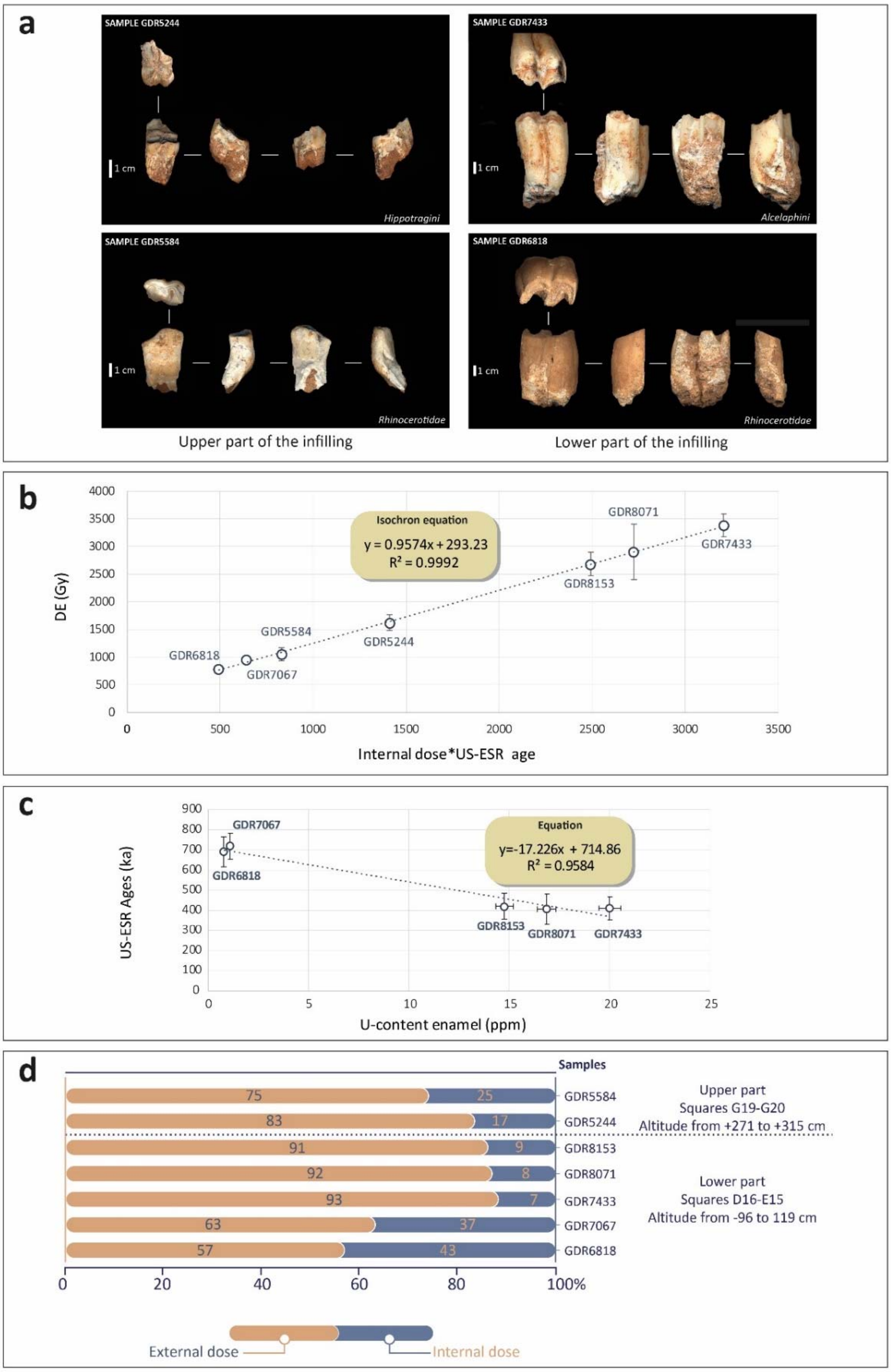

Figure S3. US-ESR dating.

a, Photos of sampled teeth for US-ESR dating (Photos by E. Ben Arous). **b**, Isochron age (Graph elaboration by C. Falguères and E. Ben Arous). **c**, Age versus U ppm (Graph elaboration by C. Falguères and E. Ben Arous). **d**, Percentage of external (orange) and internal (blue) doses for each GDR sample (Graph elaboration by E. Ben Arous).

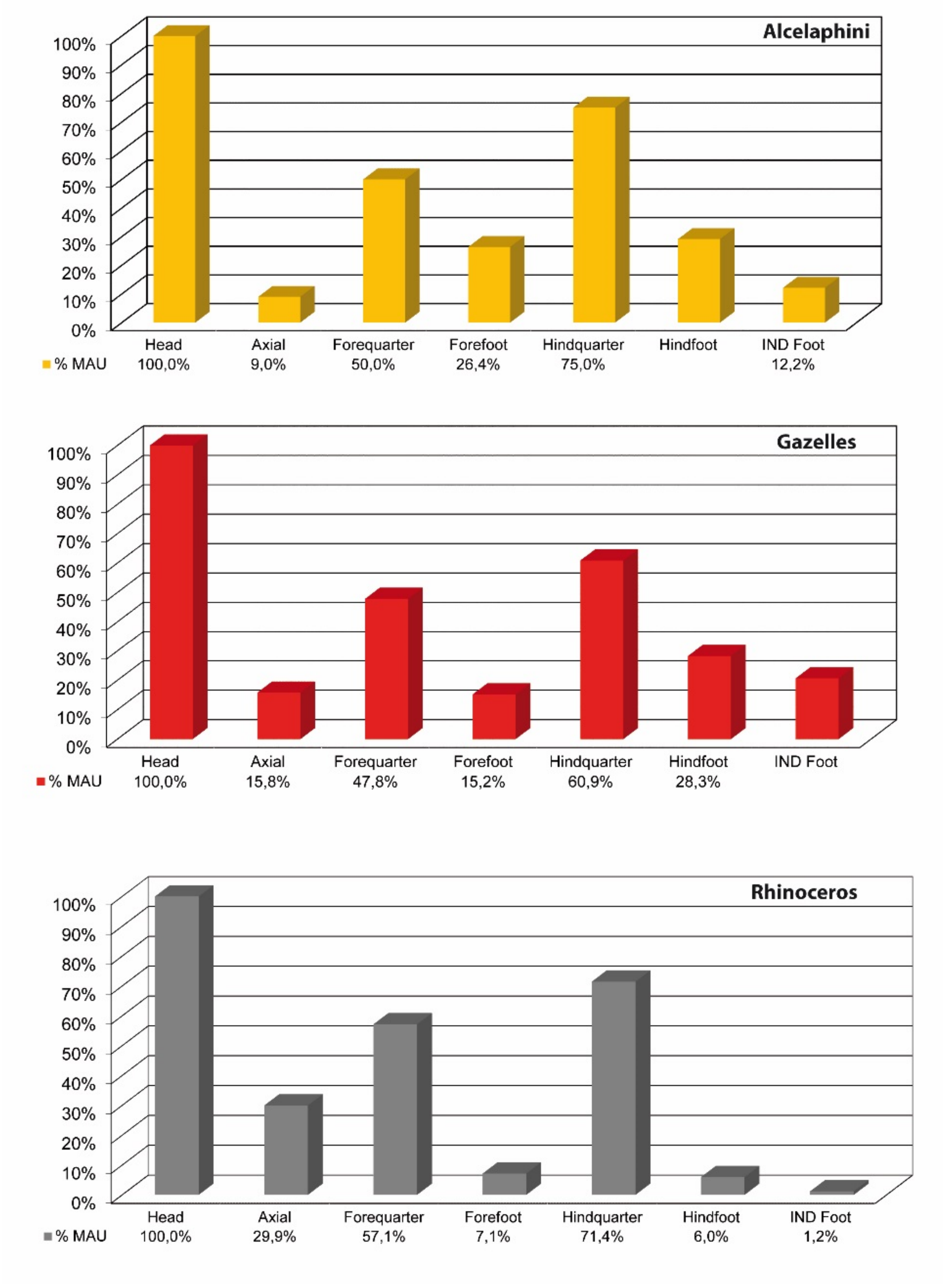

Figure S4. Skeletal profiles (% of Minimal Animal Unit) for Alcelaphini, gazelles and rhinoceros (lower set). Graphs elaboration by C. Daujeard.

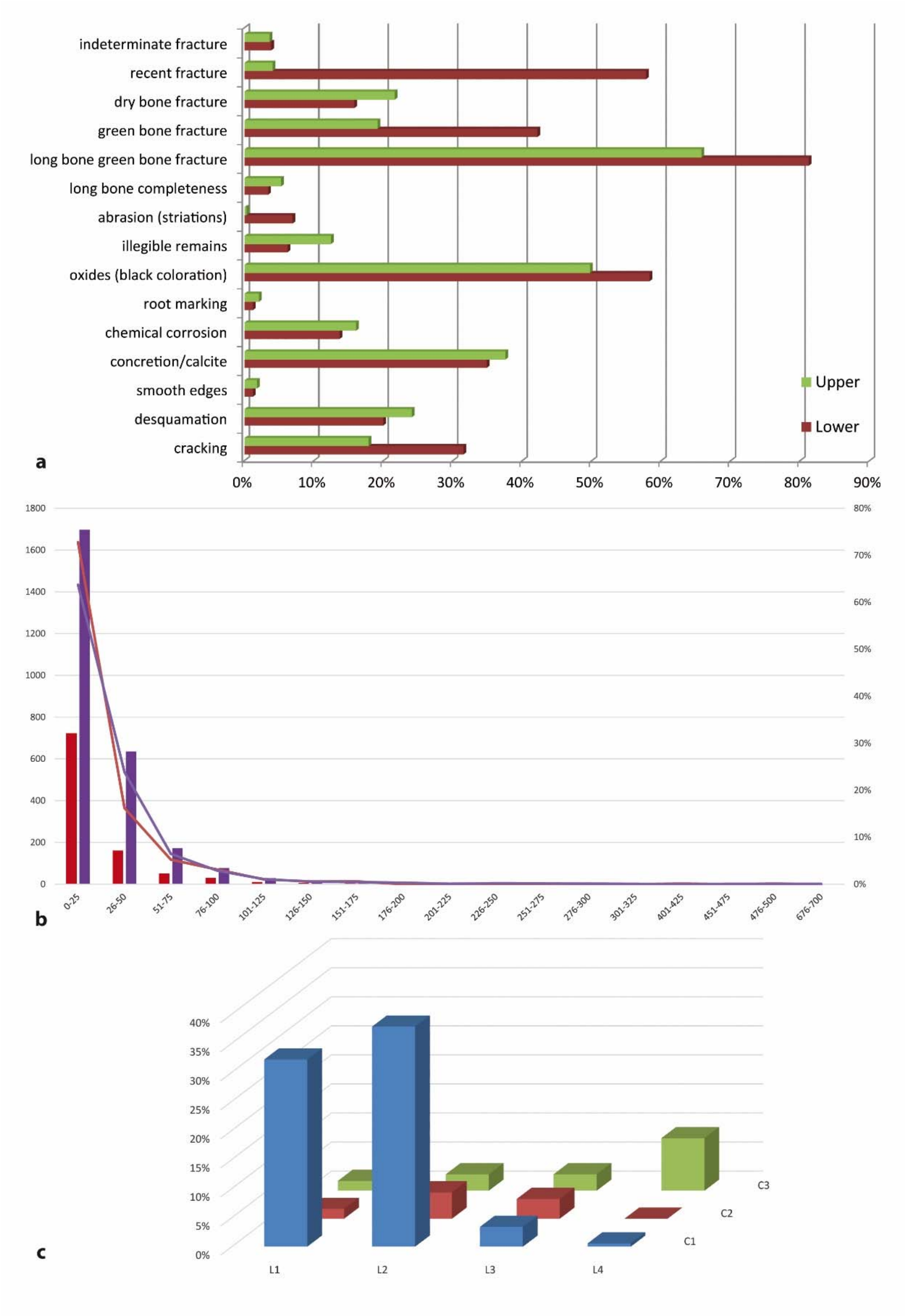

Figure S5. Preservation and fragmentation of the bone assemblages. Graphs elaboration by C. Daujeard. **a**, Various comparative indices for the two faunal series (NR lower set=986 and NR upper set=386). **b**, Size categories for the totality of bone fragments (mm) of the lower (purple) and upper (red) units (NR lower units=2662; NR upper units=993). **c**, Length and circumference for ungulate long bone fragments from the lower units (NR=177).

SUPPORTING INFORMATION (TABLES)

sample	square	unit	z (cm)	U ppm				$^{234}U/^{238}U$				$^{230}Th/^{234}U$				$^{222}Rn/^{230}Th$				T enamel	Removed enamel*
				enamel	error	dentine	error	enamel	error	dentine	error	enamel	error	dentine	error	enamel	error	dentine	error	(µm)	(µm)
GDR6818	E15	5 (lower)	-106	0,78	0,02	81,18	1,97	1,386	0,035	1,503	0,03	0,957	0,038	**1,259**	***0,044***	0,75	0,20	0,57	0,20	2820	85/49
GDR7067	*D16*	*5 (lower)*	*-96*	*1,11*	*0,04*	*85,09*	*1,81*	*1,316*	*0,046*	*1,571*	*0,027*	*1,002*	*0,046*	***1,053***	***0,036***	0,76	0,20	0,52	0,20	2684	39/72
GDR7433	*D16*	*5 (lower)*	*-108*	*20,01*	*0,55*	*58,32*	*1,15*	*1,447*	*0,026*	*1,472*	*0,01*	***1,049***	***0,042***	***1,28***	***0,043***	0,70	0,20	0,52	0,20	774	111/95
GDR8071	D16	5 (lower)	-113	16,88	0,46	63,14	1,33	1,392	0,024	1,511	0,011	0,939	0,037	**1,026**	***0,037***	0,76	0,20	0,50	0,20	900	98/87
GDR8153	*E15*	*5 (lower)*	*-119*	*14,76*	*0,44*	*74,39*	*2,04*	*1,373*	*0,033*	*1,422*	*0,033*	*0,918*	*0,039*	***1,243***	***0,048***	0,70	0,20	0,54	0,20	705	46/105
GDR5244	G19	2 (upper)	315	5,65	0,29	79,76	1,29	1,323	0,091	1,594	0,011	**1,114**	*0,059*	0,669	0,015	0,76	0,20	0,76	0,20	840	60/90
GDR5584	G20	2 (upper)	271	3,27	0,12	82,77	1,32	1,170	0,039	1,381	0,005	1,016	*0,050*	1,092	*0,036*	0,85	0,20	0,60	0,20	1970	140/40

Table S1. U content in ppm and isotopic ratios of the samples of GDR cave. The $^{222}Rn/^{230}Th$ values are obtained by combining alpha (or ICPMS) and gamma data (Bahain et al., 1992). Error range are given at one sigma for alpha and two sigma for ICPMS analyses (italics). In bold, values beyond secular equilibrium. The initial (T) and removed enamel thickness are used for the age calculation (*the first number corresponds to the enamel subtracted in the dentine-enamel side; the second number to the sediment-enamel side).

sample	square	unit	z (cm)	identification	DE (Gy)	(β + γ) sed + cosmic dose (µGy/a)	(α+β) internal dose (µGy/a)	Total dose rate (µGy/a)	p or n-value		US-ESR ages (ka)
									enamel	dentine	
GDR6818	E15	5 (lower)	-106	Rhinocerotidae (M/1-2)	784 ± 45	449 ± 38	685 ± 100	1134 ± 127	-0,54 ± 0,05	*-0,0019 ± 0,0002*	691 ± 75
GDR7067	D16	5 (lower)	-96	Rhinocerotidae (M/3)	947 ± 52	448 ± 38	867 ± 92	1315 ± 122	-0,72 ± 0,02	*-0,0014 ± 0,0002*	720 ± 64
GDR7433	D16	5 (lower)	-108	Alcelaphini (M1-2/)	3382 ± 209	508 ± 38	7702 ± 1152	8210 ± 1164	*-0,0030 ± 0,0005*	*-0,0034 ± 0,0006*	412 ± 57
GDR8071	D16	5 (lower)	-113	Alcelaphini (M3/)	2898 ± 502	497 ± 38	6605 ± 1418	7102 ± 1426	-0,73 ± 0,07	*-0,0029 ± 0,0004*	408 ± 74
GDR8153	E15	5 (lower)	-119	Alcelaphini (M1/)	2678 ± 215	506 ± 38	5854 ± 974	6360 ± 1015	-0,66 ± 0,07	*-0,0033 ± 0,0006*	421 ± 65
GDR5244	G19	2 (upper)	315	Hippotragini (M1-2/)	1615 ± 142	473 ± 38	2622 ± 399	3095 ± 409	*-0,0024 ± 0,0004*	1,09 ± 0,31	522 ± 65
GDR5584	G20	2 (upper)	271	Rhinocerotidae (P/2)	1046 ± 116	434 ± 38	1474 ± 203	1908 ± 222	*-0,0021 ± 0,0003*	*-0,0022 ± 0,0003*	548 ± 56

Table S2. Equivalent dose (DE), annual dose rates, p and n-values the latter are in italics, US-ESR ages estimation (presented at 1σ confidence level) of GDR samples. The annual dose rate components presented are: internal dose rate (α+β of the enamel); external dose rate (β contribution from the dentine, γ contribution from sediment) and cosmic dose rates.

Taxa (LUs)	NISPt	%NISPt	MNI	%MNI	Age categories
Hystrix cristata	18	2.7%	2	4,9%	
**RODENTIA**	**18**	**2.7%**	**2**	**4,9%**	
Felis sp.	2	0.3%	1	2.4%	
Panthera sp.	1	0.1%	1	2.4%	
Crocuta sp.	8	1.2%	3	7.3%	1Y, 1PA, 1OA
Hyaenidae indet.	5	0.7%			
Lupulella mohibi	30	4.5%	4	9.8%	3PA, 1OA
Ursus bibersoni	2	0.3%	1	2.4%	1OA
Mellivora capensis	3	0.4%	1	2.4%	
**CARNIVORA**	**51**	**7.6%**	**11**	**26.8%**	
**PROBOSCIDEA**	**0**	**0.0%**			
Ceratotherium mauritanicum	108	16.1%	7	17.1%	2J, 1YA, 1PA, 1OA, 2S
Equus cf. *mauritanicus*	9	1.3%	1	2.4%	1YA
**PERISSODACTYLA**	**117**	**17.4%**	**8**	**19.5%**	
Phacochoerus africanus	0	0.0%			
Camelus thomasi	1	0.1%	1	2.4%	1S
Gazella cf. *atlantica* - S1-2	164	24.4%	6	14.6%	2J, 1YA, 1PA, 1OA, 1S
Redunca - S2	0	0.0%			
Alcelaphini (cf. *Parmularius*) - S2-3	205	30.5%	8	19.5%	2J, 2YA, 1PA, 2OA, 1S
Connochaetes sp. - S3	4	0.6%	1	2.4%	
Oryx sp. - S3	0	0.0%			
Bovini (cf. *Pelorovis*) - S4	18	2.7%	2	4.9%	1J, 1OA
Bovidae indet S2	62	9.2%	**18**	**43.9%**	
Bovidae indet S3	17	2.5%			
Bovidae indet S3-4	8	1.2%			
**ARTIODACTYLA**	**479**	**71.3%**			
Homo sp.	1	0.1%	1	2.4%	1PA
Theropithecus oswaldi	1	0.1%	1	2.4%	
**PRIMATES**	**2**	**0.3%**	**2**	**4.9%**	
**LAGOMORPHA**	**5**	**0.7%**			
**NISPt/MNI**	**672**	**100.0%**	**41**	**100.0%**	
**NISPa**	746				
**NRrec**	930				
**NRT**	2873				
**Identification index**	26.0%				
**Isolated teeth**	137				
**Bone destruction index**	18.4%				
**Illegibility**	6.2%				
**Coprolites**	11				
**NRrec bones**	822				
**NRtaph**	986				
**Long bone epiph/diaph ratio**	0.4				

Table S3. Faunal spectrum (small- to large-sized mammals) and bone preservation indices for the lower units. NISPa: Number of anatomical identified specimens; NISPt: Number of taxonomical identified specimens; MNI: Minimum number of individuals; NRT: Number of total remains; NRrec: Number of recorded remains; NRtaph: Number of decipherable bone remains analyzed for the bone surface modifications; J: Juvenile; YA: Young adult; PA: Prime adult; OA: Old adult; S: Senile.

Taxa (UUs)	NISPt	%NISPt	MNI	%MNI	Age categories
Hystrix cristata	12	6.2%	2	6.7%	
**RODENTIA**	**12**	**6.2%**	**2**	**6.7%**	
Felis sp.	3	1.5%	1	3.3%	
Hyaenidae indet.	13	6.7%	1	3.3%	1PA
Lupulella mohibi	19	9.8%	2	6.7%	2PA
Ursus bibersoni	1	0.5%	1	3.3%	1OA
Mellivora capensis	1	0.5%	1	3.3%	1Y
**CARNIVORA**	**37**	**19.1%**	**6**	**20.0%**	
**PROBOSCIDEA**	**1**	**0.5%**	**1**	**3.3%**	
Ceratotherium mauritanicum	37	19.1%	4	13.3%	1J, 1PA, 1OA, 1S
Equus cf. mauritanicus	7	3.6%	1	3.3%	1PA
**PERISSODACTYLA**	**44**	**22.7%**	**5**	**16.7%**	
Phacochoerus africanus	1	0.5%	1	3.3%	1PA
Camelus thomasi	3	1.5%	1	3.3%	1YA
Gazella cf. *atlantica* - S1-2	43	22.2%	5	16.7%	1J, 1YA, 1PA, 1OA, 1S
Redunca - S2	1	0.5%	1	3.3%	1J
Alcelaphini (cf. *Parmularius*) - S2-3	14	7.2%	2	6.7%	1J, 1YA
Connochaetes sp. - S3	1	0.5%	1	3.3%	
Oryx sp. - S3	1	0.5%	1	3.3%	
Bovini (cf. *Pelorovis*) - S4	8	4.1%	1	3.3%	1S
Bovidae indet S2	6	3.1%		0.0%	
Bovidae indet S3	8	4.1%		0.0%	
Bovidae indet S3-4	3	1.5%		0.0%	
**ARTIODACTYLA**	**89**	**45.9%**	**13**	**43.3%**	
Homo sp.	0	0.0%		0.0%	
Theropithecus oswaldi	1	0.5%	1	3.3%	
**PRIMATES**	**1**	**0.5%**	**1**	**3.3%**	
**LAGOMORPHA**	**10**	**5.2%**	**2**	**6.7%**	
**NISPt**	**194**	**100.0%**	**30**	**100.0%**	
**NISPa**	237				
**NRrec**	356				
**NRT**	1097				
**Identification index**	21.6%				
**Isolated teeth**	49				
**Bone destruction**	20.7%				
**Illegibility**	12.4%				
**Coprolites**	14				
**NRrec bones**	334				
**NRtaph**	386				
**Long bone epiph/diaph ratio**	0.57				

Table S4. Faunal spectrum (small- to large-sized mammals) and bone preservation indices for the upper units. NISPa: Number of anatomical identified specimens; NISPt: Number of taxonomical identified specimens; MNI: Minimum number of individuals; NRT: Number of total remains; NRrec: Number of recorded remains; NRtaph: Number of decipherable bone remains analyzed for the bone surface modifications; J: Juvenile; YA: Young adult; PA: Prime adult; OA: Old adult; S: Senile.

stages	1	2	3	n total	NR	% n total
**cracking**	257	47	7	311	986	31.5%
**desquamation**	119	61	17	197	986	20.0%
**smooth edges**	9	2	1	12	986	1.2%
**concretion/calcite**	251	77	16	344	986	34.9%
**chemical corrosion**	102	23	10	135	986	13.7%
**root marking**	10	2		12	986	1.2%
**oxides (black coloration)**	544	29	2	575	986	58.3%
**illegible remains**				61	986	6.2%
**abrasion (striations)**				64	925	6.9%
**bone completeness (all bones)**				127	2737	4.6%
**bone completeness**				117	986	11.9%
**long bone completeness (ungulates)**				10	293	3.4%
**green bone fracture**				416	986	42.2%
**green bone fracture associated with carnivore marks**				88	416	21.2%
**green bone fracture associated with rodent marks**				43	416	10.3%
**green bone fracture associated with percussion marks**				15	416	3.6%
**dry bone fracture**				156	986	15.8%
**recent fracture**				570	986	57.8%
**indeterminate fracture**				38	986	3.9%
**green bone fracture (ungulate long bones)**				238	293	81.2%

Table S5. Bone preservation and fragmentation for the lower units.

stages	1	2	3	n total	NR	% n total
**cracking**	59	7	3	69	386	17.9%
**desquamation**	39	38	16	93	386	24.1%
**smooth edges**	7			7	386	1.8%
**concretion/calcite**	73	48	24	145	386	37.6%
**chemical corrosion**	52	10		62	386	16.1%
**root marking**	6	2		8	386	2.1%
**oxides (black coloration)**	148	37	7	192	386	49.7%
**illegible remains**				48	386	12.4%
**abrasion (striations)**				1	338	0.3%
**bone completeness (all bones)**				57	1048	5.4%
**bone completeness**				47	386	12.2%
**long bone completeness (ungulates)**				2	38	5.3%
**green bone fracture**				74	386	19.2%
**green bone fracture associated with carnivore marks**				16	74	21.6%
**green bone fracture associated with rodent marks**				1	74	1.4%
**green bone fracture associated with percussion marks**				3	74	4.1%
**dry bone fracture**				25	386	3.6%
**recent fracture**				286	386	65.8%
**indeterminate fracture**				14	386	3.6%
**green bone fracture (ungulate long bones)**				25	38	65.8%

Table S6. Bone preservation and fragmentation for the upper units.

**Taxa**	NRdig	NRcarn	NRhys	NRcut	NRhum	NRperc	NR
Hystrix cristata							3
Felis sp.							2
Panthera sp.							1
Hyaenidae		1 (16.7%)	3 (50%)				6
Lupulella mohibi	2	4 (19%)	1 (4.8%)				21
Ursus bibersoni							2
Mellivora capensis							3
Ceratotherium mauritanicum		16 (29.6%)	14 (25.9%)				54
Equus cf. *mauritanicus*		1 (11.1%)	3 (33.3%)	2 (22.2%)		2 (22.2%)	9
Camelus thomasi							0
Gazella cf. *atlantica* - S1-2	12	25 (17.5%)	12 (8.4%)	4 (2.8%)	1 (0.7%)	3 (2.1%)	143
Alcelaphini (cf. *Parmularius*) - S2-3	5	41 (23.3%)	30 (17%)	8 (4.5%)	1 (0.6%)	6 (3.4%)	176
Connochaetes sp. - S3	1	2 (50%)	2 (50%)	1 (25%)			4
Bovini (cf. *Pelorovis*) - S4		2 (14.3%)	3 (21.4%)				14
Bovidae indet S2-3	7	21 (25.9%)	6 (7.4%)	5 (6.2%)			81
Bovidae indet S3-4		3 (50%)	3 (50%)		1 (16.7%)		6
Homo sp.							0
Theropithecus oswaldi			1				1
Leporidae							5
**NISP**	27	116 (21.8%)	78 (14.7%)	20 (3.8%)	3 (0.6%)	11 (2.1%)	531
**IND**	45	99 (4.7%)	37 (1.7%)	8 (0.4%)	1 (0.05%)	5 (0.2%)	2128
**Total**	72	215 (8.1%)	115 (4.3%)	28 (1.1%)	4 (0.2%)	16 (0.6%)	2659

Table S7. Bone surface modifications for the lower units.

NRdig: Number of digested remains; NRcarn: Number of carnivore tooth-marked remains; NRhys: Number of porcupine tooth-marked remains; NRcut: Number of cutmarked specimens; NRhum: Number of human tooth-marked specimens; NRperc: Number of percussed remains; NR: Number of observed (readable) bone remains.

**Taxa**	NRdig	NRcarn	NRhys	NRcut	NRperc	NR
Hystrix cristata						2
Felis sp.						2
Hyaenidae						12
Lupulella mohibi		2 (18.2%)				11
Ursus bibersoni		1				1
Mellivora capensis						1
Proboscidean		1			1	1
Ceratotherium mauritanicum		5 (22.7%)				22
Equus cf. *mauritanicus*		0		1 (25%)	1 (25%)	4
Camelus thomasi	1	1				1
Gazella cf. *atlantica* - S1-2	3	4 (12.9%)	1 (3.2%)	2 (6.3%)		32
Alcelaphini (cf. *Parmularius*) - S2-3		1 (10%)		3 (33.3%)		9
Bovini (cf. *Pelorovis*) - S4		2 (40%)				5
Bovidae indet S2-3		1 (9.1%)		1 (9.1%)		11
Bovidae indet S3-4						2
Theropithecus oswaldi		1				1
Leporidae						10
**NISP**	4	19 (15%)	1 (0.8%)	7 (5.5%)	2 (1.6%)	127
**IND**	4	25 (2.9%)	1 (0.1%)	2 (0.2%)	1 (0.1%)	849
**Total**	8	44 (4.5%)	2 (0.2%)	9 (0.9%)	3 (0.3%)	976

Table S8. Bone surface modifications for the upper units.

NRdig: Number of digested remains; NRcarn: Number of carnivore tooth-marked remains; NRhys: Number of porcupine tooth-marked remains; NRcut: Number of cutmarked specimens; NRhum: Number of human tooth-marked specimens; NRperc: Number of percussed remains; NR: Number of observed (readable) bone remains.

NR	Series number	Units	Species	Skeletal elements	Bone category	Bone portion	Circumference	Length (mm)	Anatomical position of cut marks	Number of cut marks	Type	Muscles / Tendons	Dimensions, orientation, relationship	Morphological criteria	Orientation with respect to the major axis of the bone	Interpretation	Green bone fractures	Associated marks
1	7070	LUs	Equus mauritanicus	radius-ulna	LB	medial shaft	C1	108	lateral side	>40	Incisions	extensor digitorum muscle	short-medium-very short / very thin-thin-wide / deep-shallow-superficial / straight / parallel-non-parallel	fork-shaped ends, shoulder effects, microstriations	oblique	defleshing	1	percussion marks
1	7975	LUs	Equus mauritanicus	femur	LB	proximal shaft	C2	94	internal face of the greater trochanter	6	Incisions	gluteus medius muscle	long / very thin / shallow / straight / parallel		parallel	defleshing	1	percussion marks
1	7146	LUs	Gazella sp.	femur	LB	medial-distal shaft	C1	118	lateral side	>10	Incisions	vastus intermedius muscle	long / very thin-thin / shallow / straight / parallel	fork-shaped ends, microstriations	parallel	defleshing	1	percussion marks
	7146	LUs	Gazella sp.	femur	LB	medial-distal shaft	C1	118	above the supracondylar fossa	2	Incisions	gastrocnemius muscle	short / very thin-thin / shallow / straight / parallel		oblique	defleshing	1	percussion marks
1	7415	LUs	Gazella sp.	pelvis	FB	ilium	C1	80	external side	5	Incisions	gluteus maximus muscle (deep bundle)	medium-short / wide-thin / deep-shallow / straight-curved / parallel		oblique	defleshing	1	
1	7491	LUs	Gazella sp.	humerus	LB	distal shaft	C1	60	lateral epicondyle	5	Incisions	extensor carpi radialis muscle	medium / very thin / shallow / straight / parallel	shoulder effects	perpendicular	defleshing / disarticulation	1	
	7491	LUs	Gazella sp.	humerus	LB	distal shaft	C1	60	above the olecranon fossa	5	Incisions	anconeus muscle	medium / thin / deep-shallow / straight / overlapped	shoulder effects	oblique-parallel	disarticulation	1	
1	7527	LUs	Gazella sp.	metatarsal	LB	medial-proximal shaft	C2	69	along the posterior gutter	> 10	Incisions	flexor digitorum longus tendon	short / very thin-thin / superficial-shallow / straight-curved / parallel	shoulder effects, microstriations	oblique	skinning	1	percussion marks
1	8085	LUs	Gazella sp.	ulna	LB	medial shaft	C1	37	indeterminate side	3	Incisions	extensor digitorum muscle	long-medium / wide /deep / straight-curved / parallel	shoulder effects	oblique	defleshing ?		
1	7652/12	LUs	Gazella sp.	thoracic vertebra	SB	spinous process	C3	broken	indeterminate side	2	Incisions	spinalis thoracis (dorsi) muscle	long-short / wide-thin /deep-shallow / straight / non-parallel		oblique-parallel	defleshing		

1	sans	LUs	Gazella sp.	pelvis	FB	ilium	C1	46	internal side (medial part, below the iliac wing)	> 10	Incisions	obturator internus muscle	medium / very thin / shallow-superficial / straight / parallel-overlapped	fork-shaped ends	oblique	defleshing		
1	6841	LUs	Alcelaphini	rib	FB	medial-proximal shaft	C3	148	external side	> 10	Incisions	obliquus externus abdominis / serratus posterior muscles	medium-short / very thin /shallow / straight / parallel-non-parallel	shoulder effects	oblique	defleshing / muscle insertions cutting		
1	6984	LUs	Alcelaphini	tibia	LB	proximal shaft	C1	41	lateral side (below the foramen)	> 30	Incisions	flexor digitorum longus muscle	medium-short / very thin / shallow / straight-sinuous / parallel	fork-shaped ends, shoulder effects, microstriations	oblique	defleshing	1	percussion marks
1	7093	LUs	Indeterminate bovid S2-3	indeterminate long bone	LB	medial shaft	C1	38	indeterminate side	> 10	Incisions	indeterminate	long / very thin / shallow / straight-sinuous / parallel	fork-shaped ends, shoulder effects, microstriations	oblique	defleshing	1	
1	7159	LUs	Alcelaphini cf. Connochaetes	metacarpal	LB	medial shaft and proximal end	C3	73	anterior side (proximal shaft)	4	Incisions	palmar interossei / extensor digitorum muscles	very long-long / very thin /deep-shallow / straight-curved / parallel-non-parallel	fork-shaped ends, shoulder effects, microstriations	oblique	defleshing / disarticulation	1	
	7159	LUs	Alcelaphini cf. Connochaetes	metacarpal	LB	medial shaft and proximal end	C3	73	medial side (proximal shaft)	7	Incisions	palmar interossei / extensor digitorum muscles	long-medium / very thin /deep-shallow / straight-curved / parallel-non-parallel	fork-shaped ends, shoulder effects, microstriations	oblique	defleshing / disarticulation	1	
1	7210	LUs	Alcelaphini	tibia	LB	proximal shaft	C1	65	anterior side (below the tibial crest)	18	Incisions	semitendinosus muscle	medium / very thin /deep / straight / overlapped		oblique	defleshing	1	
	7210	LUs	Alcelaphini	tibia	LB	proximal shaft	C1	65	anterior side (internal face of the tibial crest)	> 15	Incisions	tibialis anterior muscle	medium / thin / shallow / straight / parallel		oblique	defleshing	1	
1	7673	LUs	Alcelaphini	rib	FB	medial-proximal shaft	C3	247	medial portion (external side)	> 10	Incisions	obliquus externus abdominis / serratus posterior muscles	medium / very thin / shallow / straight / parallel	fork-shaped ends, microstriations	perpendicular	defleshing	1	human tooth-marks and percussion marks
	7673	LUs	Alcelaphini	rib	FB	medial-proximal shaft	C3	247	medial portion	> 10	Incisions	obliquus externus abdominis / serratus	medium-short / very thin / shallow / straight / parallel	fork-shaped ends, microstriations	oblique	defleshing	1	human tooth-marks and

									(external side)			posterior muscles						percussion marks
	7673	LUs	Alcelaphini	rib	FB	medial-proximal shaft	C3	247	medial portion (internal side)	> 15	Incisions	intercostalis muscles	medium-short / very thin / superficial / straight / parallel	fork-shaped ends, microstriations	perpendicular	defleshing	1	human tooth-marks and percussion marks
1	7742	LUs	Alcelaphini	tibia	LB	medial-proximal shaft	C2	93	medial and posterior sides	4	incisions	popliteus muscle	medium-short / very thin / shallow / straight-curved / parallel-non-parallel	microstriations	oblique	defleshing	1	
1	7938	LUs	Indeterminate bovid S2-3	indeterminate long bone	LB	medial shaft	C1	44	indeterminate side	6	incisions	indeterminate	medium-short / very thin / shallow / straight / parallel	shoulder effects, microstriations	oblique	defleshing	1	
1	8117	LUs	Alcelaphini	femur	LB	distal shaft	C2	67	lateral side	13	incisions	vastus intermedius muscle	medium / very thin /deep / straight / parallel	fork-shaped ends, shoulder effects	perpendicular	defleshing	1	
	8117	LUs	Alcelaphini	femur	LB	distal shaft	C2	67	posterior side (along the linea aspera)	> 20	scraping marks	vastus intermedius / adductor muscles	long / very thin / shallow / straight / parallel	fork-shaped ends, shoulder effects	oblique	scraping	1	
1	8332	LUs	Alcelaphini	humerus	LB	proximal end		59	internal face of the greater tuberosity	> 10	incisions	infraspinatus / supraspinatus muscles	medium-short / very thin / shallow / straight-sinuous / parallel	fork-shaped ends, shoulder effects, microstriations	oblique	defleshing	1	
	8332	LUs	Alcelaphini	humerus	LB	proximal end		59	internal face of the deltoid tuberosity	6	incisions	deltoideus muscle	medium-short / very thin / shallow / straight-sinuous / parallel	fork-shaped ends, shoulder effects, microstriations	oblique	shoulder disarticulation	1	
1	8412	LUs	Alcelaphini	radius	LB	medial shaft	C1	66	lateral side	> 40	incisions	obliquus extensor carpi muscle	short / very thin / superficial / straight / parallel		oblique	defleshing	1	
	8412	LUs	Alcelaphini	radius	LB	medial shaft	C1	66	lateral side	> 100	scraping marks	obliquus extensor carpi muscle	short / very thin / superficial / straight / parallel		oblique	scraping	1	
1	7343	LUs	Indeterminate	rib	FB	proximal shaft	C1	43	indeterminate side	4	incisions	indeterminate	short / thin /deep-shallow / straight-sinuous / parallel	asymetric edges, shoulder effects, microstriations	oblique	defleshing	1	
	7343	LUs	Indeterminate	rib	FB	proximal shaft	C1	43	indeterminate side	5	incisions	indeterminate	short / thin /deep-shallow /straight-sinuous / parallel	asymetric edges, shoulder	oblique	defleshing	1	

														effects, microstriations				
1	7438	LUs	Indeterminate bovid S2-3	tibia	LB	medial shaft	C1	93,5	medial side	> 20	scraping marks	flexor digitorum longus muscle	short-medium / very thin / superficial / straight / parallel		oblique	scraping	1	percussion marks
	7438	LUs	Indeterminate bovid S2-3	tibia	LB	medial shaft	C1	93,5	medial side	5	incisions	flexor digitorum longus muscle	short-medium / very thin / superficial / straight / parallel		oblique	defleshing	1	percussion marks
1	7967	LUs	Indeterminate bovid S3-4	calcaneum	SB	proximal portion	C3	55	posterior side	2	incisions	gastrocnemius / calcaneus (cap) tendons	short / thin /deep / straight / parallel		oblique	tarsal disarticulation		
1	8126	LUs	Indeterminate	indeterminate long bone	LB	diaphysis extremity	C1	101	indeterminate side	4	incisions	indeterminate	medium / very thin /deep-shallow / straight / parallel-non-parallel	shoulder effects, microstriations	oblique	defleshing	1	
	8126	LUs	Indeterminate	indeterminate long bone	LB	diaphysis extremity	C1	101	indeterminate side	4	incisions	indeterminate	medium / very thin /deep-shallow / straight / parallel-non-parallel	shoulder effects, microstriations	oblique	defleshing	1	
1	7652/83	LUs	Indeterminate	indeterminate fragment	IND	indeterminate fragment	C1	17	indeterminate side	8	incisions	indeterminate	short / very thin / shallow / straight / parallel		perpendicular	indeterminate	1	
1	7652/97	LUs	Indeterminate	indeterminate long bone	LB	medial shaft	C1	28	indeterminate side	6	incisions	indeterminate	short / very thin /deep / straight / parallel		oblique	indeterminate	1	
1	7938/19	LUs	Indeterminate	indeterminate long bone	LB	medial shaft		29	along an indeterminate crest	13	incisions	indeterminate	very short / very thin / shallow / straight / parallel		oblique	indeterminate	1	
1	sac 1/30	LUs	Indeterminate	rib	FB	indeterminate fragment		20	indeterminate side	> 10	incisions	indeterminate	short / wide-very thin /deep-shallow / straight / parallel	fork-shaped ends, shoulder effects	oblique	indeterminate		
1	5538	UUs	Equus mauritanicus	metapodial	LB	medial shaft	C2	95	posterior side	5	incisions	indeterminate	short-medium / thin / shallow / straight / parallel-non-parallel		oblique	indeterminate	1	percussion marks
1	5064	UUs	Gazella sp.	pelvis	FB	ilium		50	anterior muscle insertion surface	> 15	incisions	rectus femoris muscle	very short / very thin / shallow / straight-sinuous / parallel-non-parallel		oblique	femoral-pelvic disarticulation	1	percussion marks
	5064	UUs	Gazella sp.	pelvis	FB	ilium		50	anterior muscle	2	incisions	rectus femoris muscle	very short / very thin / shallow / straight-		oblique	femoral-pelvic disarticulation	1	percussion marks

									insertion surface				sinuous / parallel-non-parallel					
1	6437	UUs	Gazella sp.	radius	LB	medial shaft		72	indeterminate side	> 10	incisions	indeterminate	short / very thin / superficial / straight / parallel-overlapped	fork-shaped ends, shoulder effects	oblique	indeterminate	1	
	6437	UUs	Gazella sp.	radius	LB	medial shaft		72	indeterminate side	> 10	scraping marks	indeterminate	medium / very thin / superficial / straight / parallel-overlapped	fork-shaped ends, shoulder effects	oblique	scraping	1	
	6437	UUs	Gazella sp.	radius	LB	medial shaft		72	indeterminate side	1	Incisions	indeterminate	medium / very thin / superficial / straight / parallel-overlapped	fork-shaped ends, shoulder effects	perpendicular	indeterminate	1	
1	6060	UUs	Alcelaphini	rib	FB	proximal end		45	indeterminate side	4	incisions	indeterminate	medium / thin / shallow / straight / parallel	microstriations	perpendicular	indeterminate		
1	6194	UUs	Alcelaphini	rib	FB	medial shaft	C1	46	indeterminate side	> 15	incisions	indeterminate	very long-long / wide-thin /deep-shallow /straight-curved-sinuous / parallel-non-parallel	fork-shaped ends, shoulder effects, microstriations	oblique-parallel	indeterminate		
1	6229	UUs	Indeterminate bovid S2-3	indeterminate long bone	LB	medial shaft	C1	45	indeterminate side	> 10	incisions	indeterminate	short-medium / thin-very thin / shallow / straight / parallel		oblique	defleshing	1	
	6229	UUs	Indeterminate bovid S2-3	indeterminate long bone	LB	medial shaft	C1	45	indeterminate side	> 20	scraping marks	indeterminate	short-medium / thin-very thin / shallow / straight / parallel		oblique	scraping	1	percussion marks
1	6453	UUs	Alcelaphini	rib	FB	proximal shaft	C3	118	along the edge	> 10	incisions	intercostalis muscles	very short / thin / shallow / straight / parallel	microstriations	oblique	indeterminate	1	
	6453	UUs	Alcelaphini	rib	FB	proximal shaft	C3	118	external side	1	incisions	obliquus externus abdominis / serratus posterior muscles	medium / shallow / straight	microstriations	oblique	indeterminate	1	
	6453	UUs	Alcelaphini	rib	FB	proximal shaft	C3	118	external side	> 10	scraping marks	obliquus externus abdominis / serratus posterior muscles	short / superficial / straight / parallel	microstriations	parallel	scraping	1	percussion notch

1	6353	UUs	Indeterminate	indeterminate fragment		indeterminate fragment	C1	57	indeterminate side	2	incisions	indeterminate	long / very thin / shallow / sinuous / overlapped	fork-shaped ends	oblique	indeterminate	1	percussion pits
	6353	UUs	Indeterminate	indeterminate fragment		indeterminate fragment	C1	57	indeterminate side	> 20	incisions	indeterminate	short / wide-very thin /deep-superficial / straight / parallel	fork-shaped ends	oblique	indeterminate	1	percussion pits
1	6367	UUs	Indeterminate	indeterminate fragment		indeterminate fragment	C1	83	indeterminate side	> 10	scraping marks	indeterminate	long / very thin / superficial / straight / parallel		oblique	scraping		

Table S9. Description and interpretation of the cut marks by bone elements.

Size classes	Weight (kg)	Bovids[40]	Ungulates[41]	Taxa (mammals)
Size 1	0-114 kg	Bovids sizes 1-2	Size 1: Small Bovids	*Gazella, Redunca, Phacochoerus,* juvenile bovines and equids, hyenids, canids, mustelids, *Hystrix*, *Felis*, *Theropithecus*, hominins
Size 2	114-300 kg	Bovids size 3	Size 2: Medium Bovids+Small Equids	*Parmularius, Connochaetes, Oryx*, *Equus*, *Ursus, Monachus*, *Panthera*
Size 3	300-1000 kg	Bovids size 4	Size 4: Large Bovids	*Bos, Pelorovis, Camelus*, juvenile *Ceratotherium*
Size 4	> 1000 kg		Size 5: very large ungulates	Adult *Ceratotherium*

Table S10. Size and weight categories of small, middle and large-sized mammals.
